# A comprehensive engineering strategy improves potency and manufacturability of a near pan-neutralizing antibody against HIV

**DOI:** 10.1016/j.str.2025.04.016

**Published:** 2025-05-14

**Authors:** Mohammad M. Sajadi, Abdolrahim Abbasi, Zahra Rikhtegaran Tehrani, Christine Siska, Rutilio Clark, Woo Chi, Michael S. Seaman, Dieter Mielke, Kshitij Wagh, Qingbo Liu, Taylor Jumpa, Randal R. Ketchem, Dung N. Nguyen, William D. Tolbert, Brian G. Pierce, Ben Atkinson, Derrick Deming, Megan Sprague, Andrew Asakawa, David Ferrer, Yasmin Dunn, Sarah Calvillo, Rui Yin, Johnathan D. Guest, Bette Korber, Bryan T. Mayer, Alicia H. Sato, Xin Ouyang, Scott Foulke, Parham Habibzadeh, Maryam Karimi, Arash Aslanabadi, Mahsa Hojabri, Saman Saadat, Roza Zareidoodeji, Mateusz Kędzior, Edwin Pozharski, Alonso Heredia, Hegang Chen, David Montefiori, Guido Ferrari, Marzena Pazgier, George K. Lewis, Joseph G. Jardine, Paolo Lusso, Anthony DeVico

**Affiliations:** 1 Divisions of Vaccine Research and Clinical Care and Research, Institute of Human Virology, University of Maryland School of Medicine, Baltimore, MD, USA; 2 Department of Medicine, Maryland VA Healthcare System, Baltimore, MD, USA; 3 Just-Evotec Biologics, 401 Terry Avenue North, Seattle, WA, USA; 4 Center for Virology and Vaccine Research, Beth Israel Deaconess Medical Center, Harvard Medical School, Boston, MA 02115, USA; 5 Duke Human Vaccine Institute, Durham, NC, USA; 6 Key Laboratory of Developmental Genes and Human Disease, School of Life Science and Technology, Southeast University, Nanjing, China; 7 Infectious Disease Division, Department of Medicine, Uniformed Services University of the Health Sciences, Bethesda, MD, USA; 8 University of Maryland Institute for Bioscience and Biotechnology Research (IBBR), Rockville, MD, USA; 9 Department of Cell Biology and Molecular Genetics, University of Maryland, College Park, MD, USA; 10 Theoretical Biology & Biophysics, Los Alamos National Laboratory, Los Alamos, NM, USA; 11 Vaccine and Infectious Disease Division, Fred Hutchinson Cancer Center, Seattle, WA, USA; 12 Neutralizing Antibody Center, IAVI, La Jolla, CA, USA; 13 Department of Biochemistry and Molecular Biology, University of Maryland School of Medicine, Baltimore, MD, USA; 14 Division of Surgical Sciences, Duke University School of Medicine, Durham, NC, USA; 15 Department of Immunology and Microbiology, Scripps Research Institute, La Jolla, CA, USA; 16 Laboratory of Immunoregulation, National Institute of Allergy and Infectious Diseases, NIH, Bethesda, MD, USA; 17 Lead contact

## Abstract

Anti-HIV envelope broadly neutralizing antibodies (bnAbs) are alternatives to conventional antiretrovirals with the potential to prevent and treat infection, reduce latent reservoirs, and/or mediate a functional cure. Clinical trials with “first-generation” bnAbs used alone or in combination show promising antiviral effects but also highlight that additional engineering of “enhanced” antibodies will be required for optimal clinical utility, while preserving or enhancing Current Good Manufacturing Practices (cGMP) manufacturing capability. Here, we report the engineering of an anti-CD4-binding site (CD4bs) bnAb, N49P9.3. Through a series of rational modifications, we produced a variant, N49P9.6-FR-LS, that demonstrates enhanced potency, superior antiviral activity in combination with other bnAbs, low polyreactivity, and longer circulating half-life. Additional engineering for manufacturing produced a final variant, eN49P9, with properties conducive to cGMP production. Overall, these efforts demonstrate the feasibility of developing enhanced anti-CD4bs bnAbs with greatly improved antiviral properties as well as potential translational value.

## In brief

Sajadi et al. describe the engineering of an anti-CD4bs HIV bnAb with significant breadth, enhanced neutralization, potent ADCC, low polyreactivity, long half-life, and superior activity in combination therapy. Further engineering produced eN49P9, which is conducive to cGMP manufacture. These findings support the clinical potential of next-generation bnAbs for HIV.

## Graphical Abstract

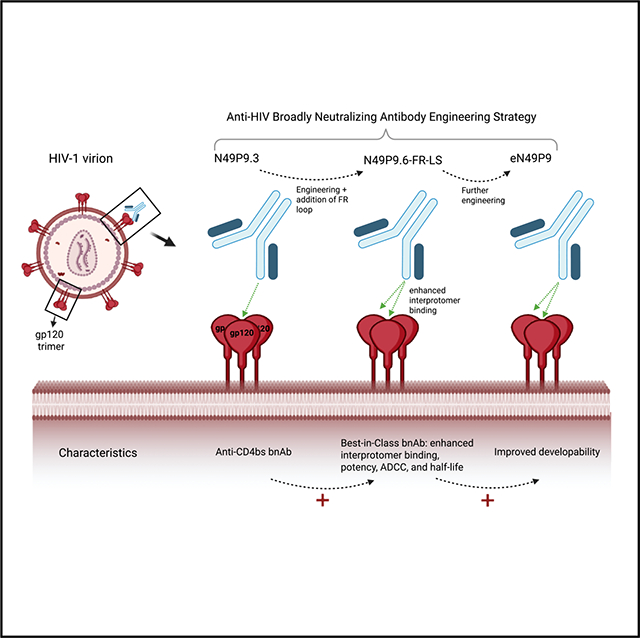

## INTRODUCTION

Currently, small-molecule antiretroviral agents (ARVs) are the only available resources for the prevention and treatment of AIDS. Although highly effective, these drugs present noteworthy limitations including differential penetration into tissue sanctuaries such as the brain, numerous side effects relevant to lifelong administration, and the inability to provide long-term, drug-free remission or functional cure. Advances in newer integrase strand transfer inhibitors provide superior efficacy and less frequent delivery compared to reverse transcriptase inhibitors but present potential long-term safety concerns regarding links to diabetes and metabolic syndrome.^1–6^ The capsid inhibitor lenacapavir offers injections every 6 months; its only drawbacks to date are the lag time prior to full efficacy and injection site reactions.^7^

Broadly neutralizing antibodies (bnAbs) against the HIV envelope (Env) trimer are promising alternatives or ARV-complementing agents for HIV prevention and treatment, reduction of latent virus reservoirs, and/or cure strategies. Such antibodies arise in a small percentage of individuals who are chronically infected with HIV,^8,9^ often after accumulating substantial somatic hypermutation.^10^ BnAbs target an array of conserved sites (reviewed in the study by Haynes and Mascola^11^) within the Env trimer: the apex comprising gp120 V1/V2 amino acids and glycans; the gp120 V3 loop domains comprising the N332-glycan; high-mannose regions in the gp120 outer domain^12^; the CD4-binding site (CD4bs) of gp120^13^; the membrane-proximal region of gp41 (reviewed in the study by Kwong and Mascola^14^); and a combination of gp120-gp41 interface sequences.^15^ Although each class has specific strengths and limitations, a general advantage of bnAbs is that they lack toxicity and may act against HIV through multiple effector mechanisms including direct virus inactivation as well as Fc-mediated phagocytosis and killing of infected cells.^16,17^

Clinical testing of “first-generation” human bnAbs has yielded promising safety and efficacy results. The Antibody-Mediated Prevention (AMP) trial of the anti-CD4bs antibody, VRC01, found that participants that received infusions of recombinantly produced VRC01 IgG were protected against more sensitive viruses.^18^ However, overall efficacy was negligible as VRC01 lacked sufficient breadth to act on the majority of circulating viral strains.^18^ The recently completed TITAN trial of 3BNC117 (anti-CD4bs) and 10–1074 (anti-V3) highlighted the promise of dual bnAb treatment in eliciting long-term viral suppression.^19^ The TATELO trial of VRC01 and 10–1074 in a pediatric cohort demonstrated that dual bnAb treatment safely maintained HIV suppression for up to 24 weeks in 44% of children after cessation of conventional ARVs.^20^ Such findings suggest that bnAbs could offer a feasible strategy of ART sparing in children to avoid long-term toxicities and adherence issues. Furthermore, through a process still under investigation, bnAb treatment appears to mediate a so-called “vaccinal effect” that promotes more efficient host immune control of infection in addition to mediating neutralization.^21^ A recent trial of triple bnAb therapy reported long-term viral suppression in 42% of volunteers even after bnAb titers became undetectable.^22^ Thus, bnAb treatment could enable functional cure strategies^23^ and offer a range of treatment options that suit individual needs.^24^

These trials also highlight why full clinical value will require greater breadth and potency of action. Attempts to pre-screen for viruses resistant to current bnAbs may exclude up to 46% of volunteers.^25^ Fortunately, rational engineering of bnAbs to achieve greater potency, breadth, and pharmacokinetic (PK) characteristics, while allowing feasible Current Good Manufacturing Practices (cGMP) production, is a rational means of overcoming these limits. Here, we report such engineering of a near pan-neutralizing anti-CD4bs bnAb N49P9.3 isolated directly from the plasma of HIV elite neutralizer.^26^ We reasoned that such an antibody would be highly conducive to achieving the aforementioned improvements, boosting prospects for successful bnAb-based treatment and prevention options in HIV/AIDS. Through a series of rational modifications, we produced N49P9.6-FR-LS that demonstrates enhanced potency, superior antiviral activity in combination with other bnAbs, low polyreactivity, and longer circulating half-life. Additional engineering for manufacturing produced a final variant, eN49P9, with properties conducive to cGMP production.

## RESULTS

### Primary engineering for improved breadth, potency, and PK

As previously reported, we assembled a cohort of people living with HIV (PLWH) whose plasma demonstrated the presence of bnAbs against HIV.^26–29^ One subject (N49) harbored particularly broad and potent plasma-neutralizing activity, maintained in circulation over a 12-year period as shown by tests in multi-tier pseudovirus panels in the TZM-bl assay (not shown). N49 plasma^26^ collected in 2012 neutralized 99% of 117 pseudoviruses comprising tier 1–3 isolates in a global panel. From this subject, plasma bnAb H and L chain sequences were assembled using a B cell gene database. This exercise traced broadly neutralizing plasma activity to two families of anti-CD4bs bnAbs termed N49P7 and N49P9. Both families use immunoglobulin heavy variable 1–2 (IGHV1–2) heavy chain (subclass IgG1) and λ light chains, with deletions in CDRL1 and CDRL3, but are distinguished by use of different λ light-chain families and CDRH3 lengths.^26^ Further mining of the N49 B cell database identified other antibodies with closely matched CDRH3 clonal variants with superior neutralizing activity. One of them, designated N49P9.3, was selected for further engineering. The progression of modification and testing is summarized in the flow-chart shown in Figure S1.

All wild-type N49 P-series bnAbs carry an amino acid mutation in the first position of the heavy and/or light-chain constant regions, due to the final position of the J segment determining the first nucleotide of the codon. We previously noted that reversion of this mutation to the germline residue yielded slightly improved neutralization in several N49 P-series bNAbs. Accordingly, a P to A mutation (P1.4A CH1) in the first position of the heavy-chain constant region and an R to G mutation (R1.5G IGLC7) in the first position of the light-chain constant region were introduced in N49P9.3, yielding N49P9.6.

**Figure 1.**
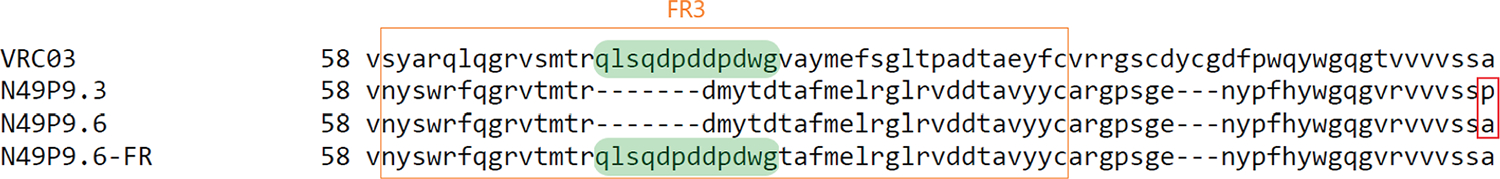
Engraftment of heavy-chain framework-3 loop from VRC03 into N49P9.6 to engineer N49P9.6-FR The amino acid differences between N49P9.3 and N49P9.6 heavy chain are shown in the red box. The heavy-chain framework-3 loops of the bnAbs are set within the orange box, and the part of VRC03 engrafted into N49P9.6 is shaded in green.

Using a previously reported design strategy,^30–32^ N49P9.6 and N49P9.3 were engrafted with a 12-residue acidic and elongated heavy-chain framework-3 loop (“FR”) of the anti-CD4bs antibody VRC03 (Figure 1) to facilitate an additional quaternary interaction with the CD4bs of an adjacent gp120 protomer in the Env trimer.^30,33^ This modification increases the neutralization potency of anti-CD4bs bnAbs and improves their overall biochemical behavior.^30–32^ The resulting variants, N49P9.6-FR and N49P9.3-FR, were then subjected to further structural and functional analyses.

**Figure 2.**
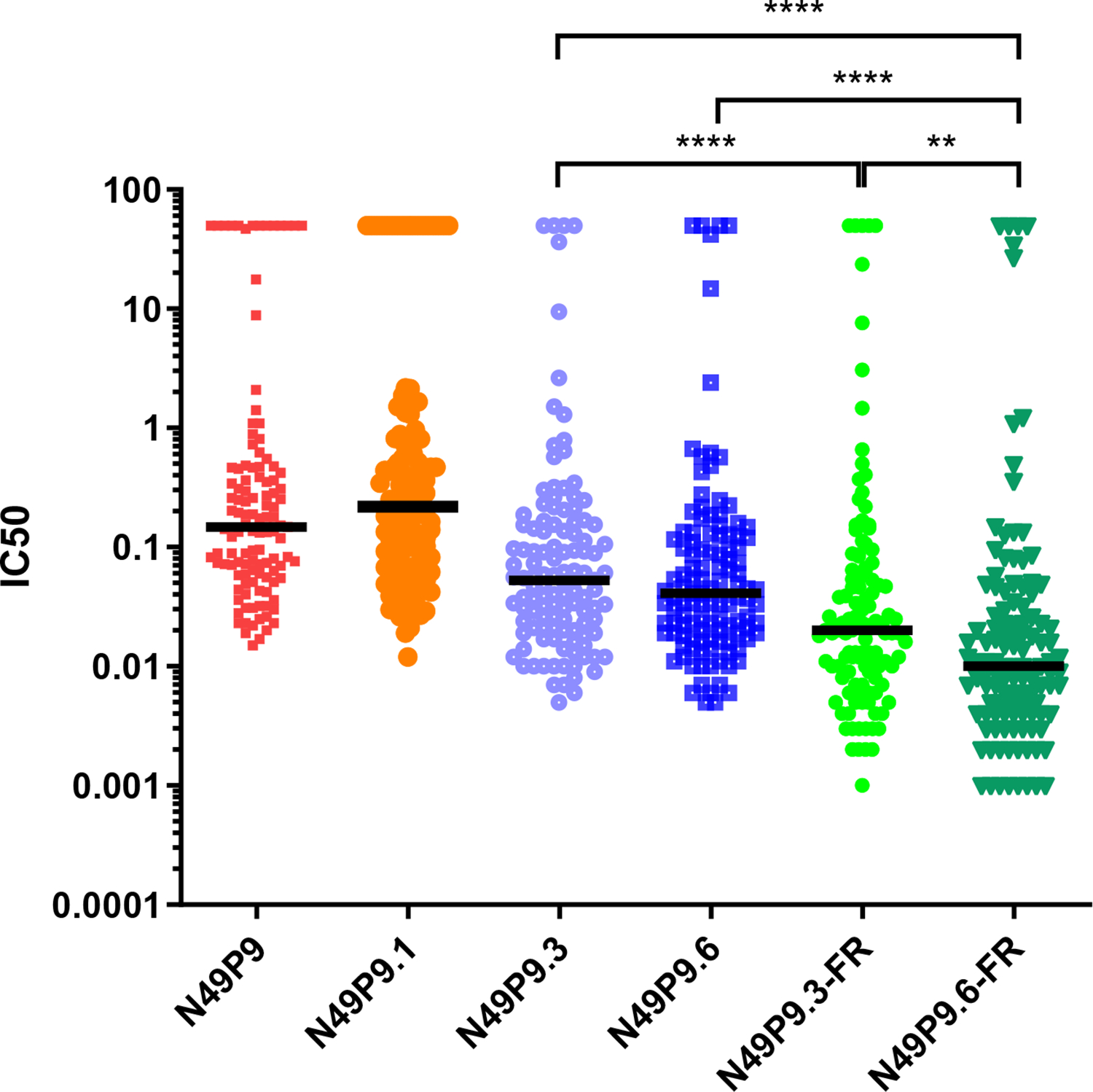
Improvement in neutralization through engineering Variants of N49P9.3 show lower IC50 values compared to parental forms in 118 multi-tier multi-clade pseudovirus panel. N49P9 and N49P9.1 (identical VDJ to N49P9 but with LC7 instead of LC2) also included for comparison. N49P9.6-FR has an IC50 and IC80 of 0.01 and 0.03 μg/mL, respectively. IC50 values between groups compared by Mann-Whitney U test: **p < 0.01; ****p < 0.0001.

Sequential engineering from N49P9.3 to N49P9.6-FR progressively improved the neutralization breadth and potency as determined against a global 118 pseudovirus panel representing multiple clades and neutralization tiers (Figure 2; Figure S2). N49P9.6-FR demonstrated 97% coverage at a median IC50 potency of 0.01 μg/mL and IC80 potency of 0.03 μg/mL. Overall, the median IC50 value for N49P9.6-FR was 5.3-fold and 4.1-fold lower than for N49P9.3 or N49P9.6, respectively (*p* < 0.0001), and 2-fold lower than N49P9.3-FR (*p* < 0.01) (Figure 2). Similarly, the median IC80 values for N49P9.6-FR were 5-fold and ~2-fold lower than for N49P9.3 or N49P9.6, respectively (*p* < 0.0001).

**Figure 3.**
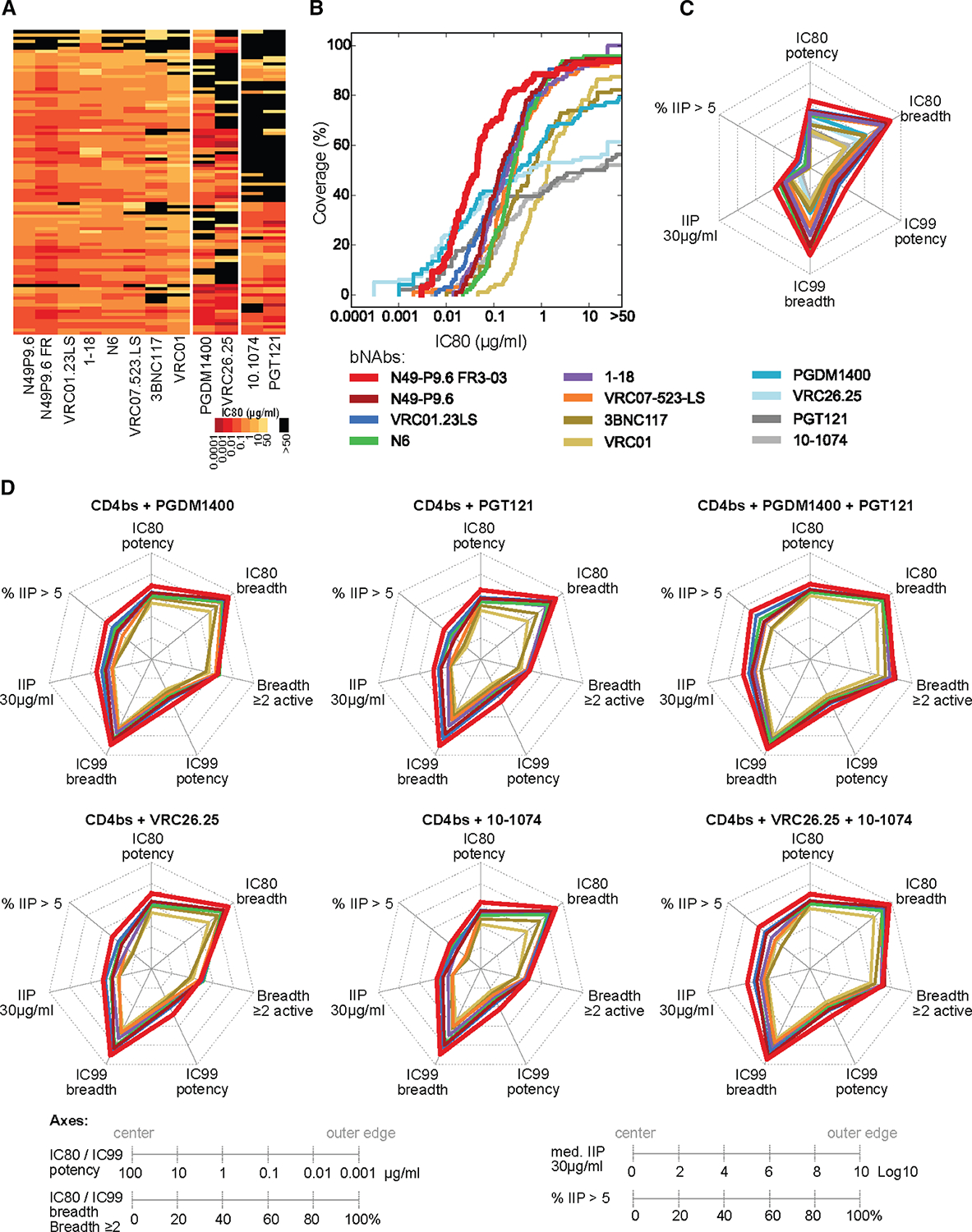
N49P9.6-FR outperforms other bnAbs individually and as part of bnAb combinations (A) Heatmap of IC80 titers for N49P9.6 and N49P9.6 FR and other best-in-class CD4bs, V2 apex, and V3 glycan bnAbs. (B) IC80 breadth-potency curves for bnAbs in (A). (C) Spiderplots showing multimetric comparison of bnAbs using IC80 breadth and potency, IC99 breadth and potency, and IIP median and breadth (IIP > 5log10). Axes ticks are defined in (D), and for each metric, radially outwards is better performance. (D) Similar to (C) except evaluating 2- and 3-bnAb combinations. Each CD4bs bnAb is combined with V2, V3, or both bnAbs, and IC80, IC99, and IIP are predicted. IC80 and IC99 breadth values were calculated at 1 μg/mL and 10μg/mL, respectively. Colors in (C) and (D) correspond to the color of the CD4bs bNAb in (B).

Given these findings, we more extensively assessed the benefits of the basic N49P9.6-FR design in three ways. First, we compared the design to other CD4bs, V2 apex, and V3 glycan bnAbs for the general qualities of breadth and potency. Data were either generated *de novo* or obtained from the CATNAP database,^34^ comprising a global neutralization panel of 96 pseudoviruses (derived from the 118 pseudovirus panel). Comparing potency (IC80) titers for individual bnAbs, N49P9.6-FR exhibited a geometric mean of 0.069 μg/mL, a 3-fold increase over the next best score for VRC01.23LS. At the benchmark potency for human protection (IC80 < 1 μg/mL set by the AMP trials), N49P9.6-FR showed the highest breadth of 88.5% (Figure 3; Table S1). In more stringent comparisons, N49P9.6-FR also exhibited the highest IC99 titers and breadth (Table S1). We also compared bnAbs for instantaneous inhibition potential (IIP) representing ≥5log10 reduction in viral replication, which was associated with clinical success of antiviral agents in HIV-1 therapeutic settings.^35^ Generally, individual bnAbs meet this bar for very few HIV variants.^36,37^ Nevertheless, N49P9.6-FR topped the list by exhibiting >5log10 IIP against 14.6% of viruses (Figure 3; Table S1).

Second, we assessed the compatibility of N49P9.6-FR to act with other bnAb classes in antibody cocktails. Such mixtures can mitigate limits in IIP and coverage seen with individual bnAbs (including N49P9.6-FR) by recognizing multiple nonoverlapping epitopes and escape variants. Neutralization potency and breadth is also higher with mixtures, particularly with respect to IIP.^36–39^ Using a Bliss-Hill model,^36^ relevant scores (IC80, IC99, and IIP) were predicted and compared among various 2- and 3-bnAb combinations. Breadth of ≥2 bnAbs active was also assessed, as this property can counter the high within-host diversity found in PLWH. As shown in Figure 3 and Tables S2 and S3, the overall performance of all combinations was uniformly superior when N49P9.6-FR was used as the anti-CD4bs component. Across metrics, the superior bivalent combination was N49P9.6-FR + PGDM1400; the superior trivalent combination was N49P9.6-FR + PGDM1400 + PGT121. The latter was predicted to neutralize 94.8% viruses at IC80 < 1 μg/mL (total bnAb concentration), 93.8% at IC99 < 10 μg/mL, and 71.9% at IIP > 5log10.

Third, we assessed the autoreactivity and polyreactivity properties of N49P9.6-FR using a clinical panel of human antigens. Several bnAbs, including those in the anti-CD4bs class, demonstrate variable levels of such reactivity,^40,41^ which may confound vivo pharmacokinetics and clinical testing. However, N49P9.6-FR tested negative for reactivity with all of these antigens (Table S4).

The strong functional performance of N49P9.6-FR *in vitro* prompted us to further modify the design to optimize circulating half-life *in vivo*. Specifically, two mutations (M428L and N434S) known to increase Fc-binding affinity for the neonatal Fc receptor (FcRn) and increase plasma half-life^42,43^ were introduced into the IgG1 Fc domain. The resulting variant, N49P9.6-FR-LS, was confirmed to preserve the neutralization profile of N49P9.6-FR (less than a 2-fold difference in median IC50 and IC80) (Figure S2). The impact of the Fc modification on functionality was further assessed in antibody-dependent cell-mediated toxicity (ADCC) assays using a panel of infectious molecular clones encoding recently circulating Env sequences obtained from placebo infections in recent clinical trials (HVTN703/HVTN704 and HVTN705). As shown in Figure S3, both variants demonstrated broad ADCC activity, although potency was discordant between the two for certain viruses.

N49P9.6-FR-LS was then tested for plasma half-life in hFcRn mice, which harbor a knockout allele of the FcRn α-chain (Fcgrt^tm1Dcr^), express the human FcRn α-chain, and are extensively used for measuring *t*_1/2_ of human monoclonal antibodies (mAbs) and Fc-engineered mutants.^44^ Importantly, the *t*_1/2_ in this model strongly correlates with *t*_1/2_ in humans, superior to non human primate (NHP), hemizygous hFcRn transgenic mice, and wild-type mouse models.^44^ As shown in Table 1, both the -FR insertion and the LS mutations prolonged the *t*_1/2_, as previously reported.^32^ Notably, N49P9.6-FR-LS had a longer *t*_1/2_ than any other CD4bs bnAbs tested, including VRC01-LS (Table 1), which has a half-life of 71 days in humans.^45^

Having established the *in vivo* PK of N49P9.6-FR-LS, *in vivo* efficacy was assessed using CD34-NSG-SGM3 and CD34-NSG-IL15 mice (reconstituted with human CD34^+^ stem cells). The latter model efficiently reconstitutes human T cells as well as natural killer (NK) cells,^46,47^ thus enabling Fc-dependent effector functions to act along with fragment antigen binding (Fab)-mediated Env inactivation.^17,46^ A recent study in reconstituted MISTRG-IL15 mice highlighted how NK cells help control HIV infection via ADCC when recruited by PGT121.^17^

**Figure 4.**
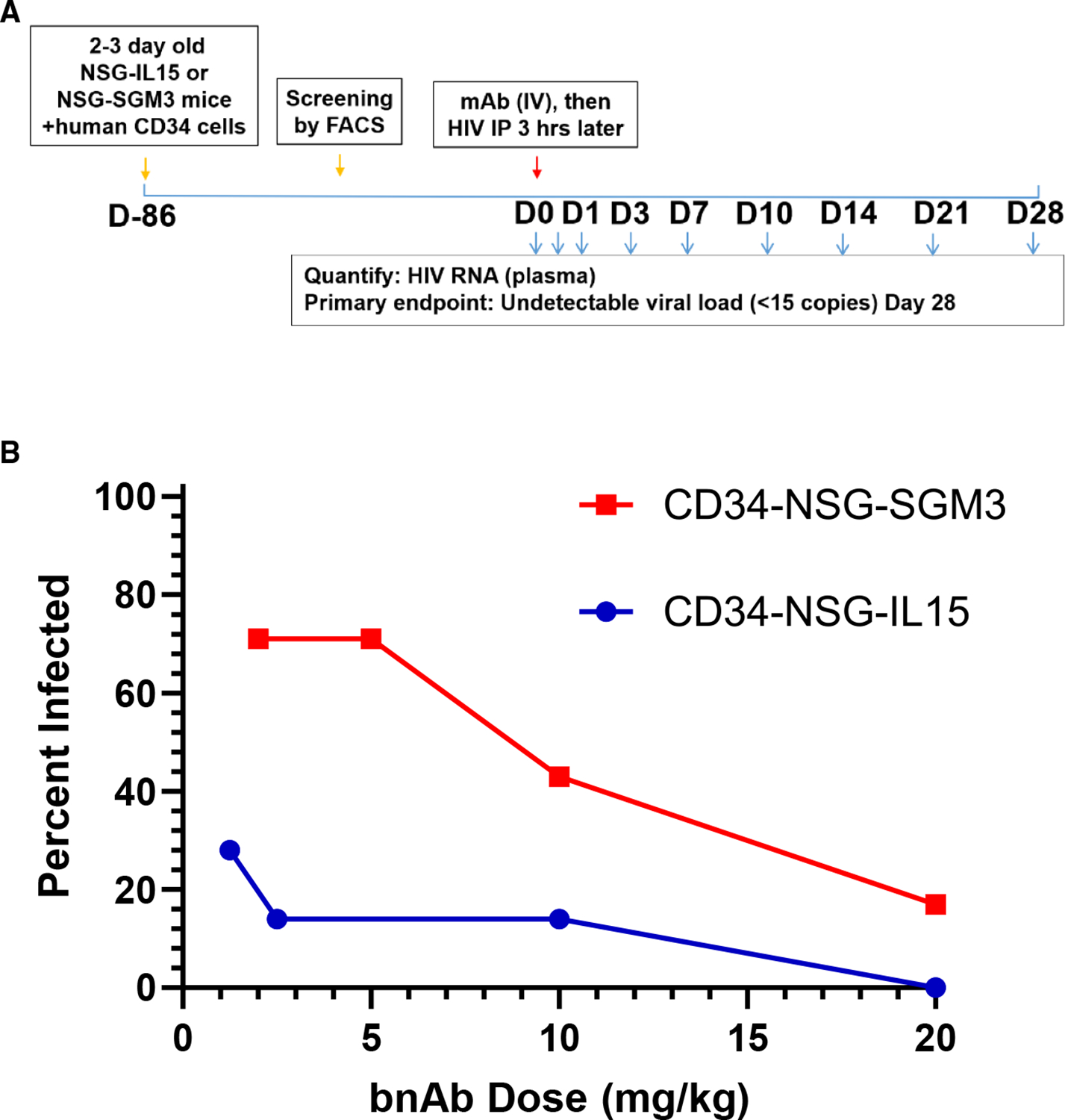
N49P9.6-FR-LS protective efficacy in CD34-NSG-SGM3 and CD34-NSG-IL15 mouse models (A) Experimental schema. Mice were injected i.p. with the indicated doses of bnAb and 3 h later challenged with HIV 1086c i.p. Plasma viremia was measured by quantitative reverse-transcription PCR (RT-qPCR) weekly to determine infection status. (B) Dose-effect curves for the bnAb measured in either mouse strain. Significantly greater protection was seen in Hu-CD34-IL15 mice (*p* < 0.0001 by logistic regression for binary outcome at week 3). All control mice injected with Synagis became infected in both mouse strains (Table S5). The experiment was repeated once with similar results.

In the experiment described further (Figure 4A), at baseline pre-challenge, the median fractions of human CD45^+^ cells in CD34-NSG-IL15 mice were 28.6 (standard deviation 11.4); in CD34-NSG-SGM3, 45.2 (standard deviation 12.2). Median fractions of CD4^+^ cells in CD34-NSG-IL15 mice were 67.4 (standard deviation 12.0); in CD34-NSG-SGM3, 49.3 (standard deviation 15.4). Median CD4+/CD8+ T cell ratios in CD34-NSG-IL15 mice were 2.1 (standard deviation 1.3); in CD34-NSG-SGM3 mice, 5.8 (standard deviation 2.9). In comparison, and as expected, fractions of CD45^+^ and CD56^+^ cells in the CD34-NSG-IL15 mice (median 11.1; standard deviation 6.3) were substantially higher than in the CD34-NSG-SGM3 strain (median 0.7; standard deviation 0.93). Within each strain, there were no significant differences in cell subset fractions between bnAb treatment and control groups (*p* > 0.05 by Kruskal-Wallis test).

Serial dilutions of bnAb were administered intraperitoneally (i.p.) (7–8 animals/dosing group). The anti-respiratory synctial virus (RSV) antibody, Synagis, served as negative control, administered to match the highest bnAb concentration tested. Prior to virus challenge, the Hu-CD34-IL15 mice were bled to determine mean bnAb plasma concentrations. Mice were challenged i.p. with the appropriate virus dose 3 h later. To rigorously test efficacy, a clade C transmitted/founder virus (1086c) with moderate sensitivity to N49P9.6-FR-LS (IC80 of 1.6 μg/mL in the TZM.bl neutralization assay format; data not shown) was used at doses previously determined to infect all untreated Hu-CD34-NSG and Hu-CD34-IL15 mice (30 and 75 TCID_50_, respectively).

Animals were tested 21 days post challenge for serial plasma viral loads or until euthanasia was required. Treated animals were scored as uninfected if plasma viremia remained undetectable at day 21. In the control groups, there was no association between infection outcome and CD34^+^ cell donor used to reconstitute the animals. The dose-effect curves showed that the 20 mg/kg dose protected 71% of NSG-SGM3 mice but 100% of the NSG-IL15 mice (Figure 4B; Table S5). The curves further reflected that the bnAb was significantly more potent in CD34-NSG-IL15 mice. The odds ratio of infection in the CD34-NSG-IL15 mice to CD34-NSG-SGM3 mice is 0.098 (*p* < 0.0001). In CD34-NSG-IL15, the groups with >85% protected animals had bnAb titers ≥10 μg/mL, corresponding to IIP values against the challenge virus ≥3.6, calculated as previously described.^35,48–50^

### Molecular basis for N49P9.6-FR activity *in vitro Crystal structures of N49P9.1, N49P9.3, and N49P9.3-FR Fabs bound to clade A/E gp120_93TH057_core*_*e*_

To define the molecular basis of Env antigen recognition by N49P9.6-FR, we began by structurally characterizing the antigen-bound complexes of other antibodies from the N49P9 lineage, specifically N49P9.1, N49P9.3, and N49P9.3-FR. Fabs of these antibodies were complexed with clade A/E gp120_93TH057_core_e_, and their structures were solved by X-ray crystallography (Figure S4; Table 2). As shown in Figure S4, N49P9.1 shares many of the characteristic features of VRC01-like IGHV1–2 antibodies that utilize their heavy-chain complementary determining region 2 (CDR H2) to mimic CD4’s interaction with gp120 including a short antiparallel β sheet between heavy-chain residues 55 to 57 and the CD4-binding loop (gp120 residues 365 to 368) and a characteristic salt bridge between heavy-chain framework residue Arg^71^ and gp120 Asp^368^.^51^ N49P9.1 has a tyrosine at heavy chain position 54 to mimic Phe^43^ of CD4 as does the VRC01-like antibody N6^52^; VRC01 and N49P7 have a glycine at position 54.^26,51^ Although clearly VRC01-like, the CDR H2 of N49P9.1 is more similar to N49P7 than to VRC01^26^ (Figure S5). Its CDR H3 is short; however, it is one amino acid shorter than VRC01, meaning that it lacks the long CDR H3 that is unique in the N49 P7 lineage of antibodies.^26,53^ Also like N49P7, N49 P9 series antibodies utilize a lambda light chain with deletions in CDR L1 and CDR L3, but their deletions in CDR L1 are one amino acid shorter than those in the N49 P7 lineage.^26^ This is in contrast to most other VRC01-like antibodies, which utilize kappa light chains with deletions in CDR L3.^54^ On the whole, N49P9.1 can be thought of as a more VRC01-like version of N49P7.

Next, we characterized N49P9.3 and its variant with -FR insertion, N49P9.3-FR. N49P9.3’s heavy and light chains are 96.8% and 96.5% identical in sequence to N49P9.1’s. Analysis of the two structures reveals that only two gp120 contact residues differ between N49P9.1 and N49P9.3, one in CDR H1 and one in CDR H2 (Figures S4B and S4C). The first, Phe^33^ in CDR H1, packs against gp120 Ala^281^ in place of Leu^33^ in N49P9.1, but with slightly better van der Waals contacts, and modulates heavy-chain Trp^50^, which also packs against gp120 Ala^281^ and forms a hydrogen bond to gp120 Asn^280^. The second Gln^64^ in CDR H2 can form hydrogen bonds with both gp120 conserved residues Arg^469^ and Asp^457^ versus Glu^64^ in N49P9.1, which can only form a hydrogen bond with Arg^469^. A Gln at position 64 is also what is seen in both N49P7 and VRC01 implying that Gln can make an important contribution to binding when placed here.^25,71^ These two amino acid changes likely account much of N49P9.3’s increase in neutralization potency and breadth relative to N49P9.1.

Structures of N49P9.3 with and without -FR allowed us to assess the framework’s role in binding monomer gp120. We were able to confirm that the insertion in N49P9.3-FR makes little direct contact to gp120 core in the N49P9.3-FR Fab-gp120_93TH057_core_e_ complex as expected; the -FR insertion is only expected to contact the adjacent gp120 protomer in the Env trimer.^32^ The only direct contact the insert makes is to two glycines in the truncated V1V2 loop in the gp120_93TH057_cor_e_ construct, which are not present in the original V1V2 loop sequence (Figure S4C).

### Cryo-EM structure of N49P9.6-FR and PGT121 Fabs bound to BG505 SOSIP.664

**Figure 5.**
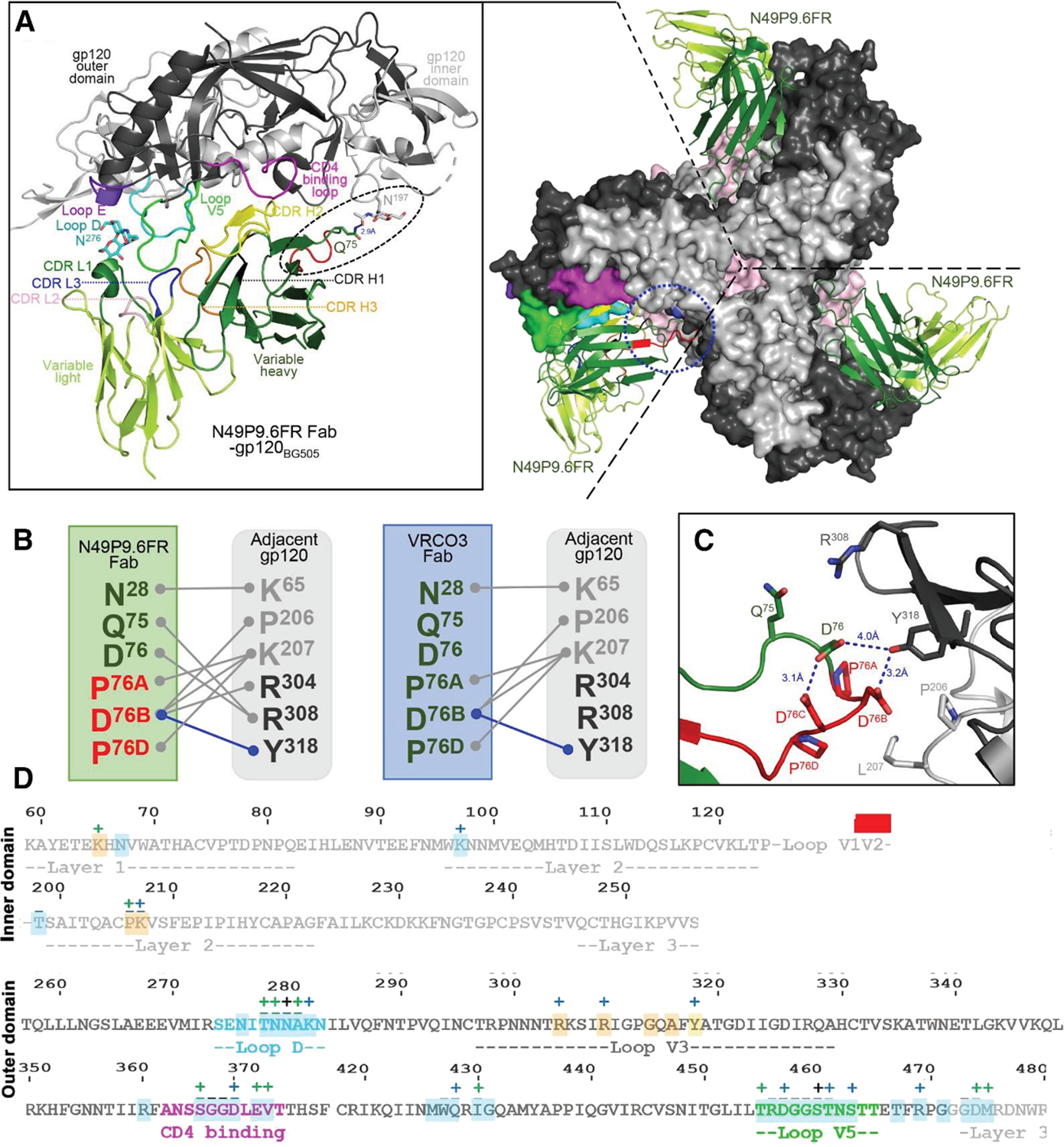
Molecular details of the Env trimer interprotomer contacts of N49P9.6-FR (A) Top view of the complex of BG505 SOSIP.664 HIV-1 Env trimer with N49P9.6-FR (the Fabs of PGT121 were omitted for clarity). The three N49P9.6-FR Fabs (the cryo-EM model was built with the variable regions of the Fabs only, dark and light green ribbon) are shown bound to the Env trimer (inner and outer gp120 domains colored light and dark gray, respectively). On the right, one N49P9.6-FR-gp120BG505 complex was extracted from the trimer to show the details of the Fab contacts to the primary gp120 protomer. One additional primary gp120 contact of N49P9.6-FR that is only seen in the Env trimer and is mediated by the region preceding the introduced frame sequence is encircled. On the bottom, a zoomed-in view depicts the contact between N49P9.6-FR with the adjacent BG505 protomer. All contact residues within a 4 Å distance criterion cutoff are shown as sticks, and H-bonds are shown as blue dotted lines. The residues of the introduced frame sequence are shown in red. (B) Network of interactions formed between N49P9.6-FR with the adjacent gp120BG505 protomer as defined by a 5 Å distance criterion cutoff. The contacts for VRC03 by the same criteria to the adjacent gp120BG505 protomer are shown for comparison (PDB ID: 6CDI). (C) A zoomed-in view depicts the contact between N49P9.6-FR with the adjacent BG505 protomer. All contact residues within a 4 Å distance criterion cutoff are shown as sticks, and H-bonds are shown as blue dotted lines. The residues of the introduced frame sequence are shown in red. (D) Epitope footprints of Fabs mapped onto the gp120 primary sequences. Contact residues are defined by a 5 Å cutoff and marked above the sequence with (+) for side chain and (−) for main chain to indicate the type of contact. Contact types are colored as following: hydrophilic (blue), hydrophobic (green), and both (black). Buried surface residues determined by PISA are shaded blue for primary gp120 contact residues and orange for adjacent gp120 contact residues.

Finally, we resolved the structure of N49P9.6-FR Fab in complex with a BG505 SOSIP.664 trimer by cryoelectron microscopy (cryo-EM) with the Fab of the chaperone antibody PGT121 (Figures 5 and 7; Table S6). Refinements of this 4.0 Å resolution structure were informed by high-resolution crystal structures of N49P9.3 and N49P9.3-FR Fabs in complexes with the gp120_93TH057_ core_e_. This operation was informative as N49P9.6 has only two amino acid differences outside of the variable region (at the beginning of the constant regions) relative to N49P9.3 and is nearly identical in both the N49P9.6-FR Fab-PGT121-BG505 SOSIP.664 and N49P9.3-FR Fab-gp120_93TH057_core_e_ complexes, with most contacts to the primary protomer in the trimer matching those observed in binding to the gp120 monomer (Figures S4B and 5C). The only exception is an additional contact of heavy-chain framework-3 residue Gln^75^ of N49P9.6-FR to the glycan on Asn^197^ of gp120 seen only in the trimer complex (Figure 5A). A similar contact was previously observed in the complex of the N49 P7 lineage N49P6 Fab with BG505 SOSIP.664, where framework Asp^72^ of N49P6 contacted the gp120 glycan on Asn^197^.^53^ Asn^197^ is absent in the gp120_93TH057_core_e_ complexes because of the V1V2 loop truncation. Other minor differences in contacts between the gp120 core and SOSIP structures are due to a lack of sequence identity at six contact residues (371, 430, 460–461, and 474–475) (Figures S4B and 5D). However, the changes are conservative in nature and do not disrupt N49P9.6-FR binding. The total buried surface area (BSA) for the N49P9.3-FR Fab-gp120_93TH057_core_e_ structure is 2,491 Å^2^ (1,254 Å^2^ for gp120 and 1,237 Å^2^ for Fab) while the total BSA for the primary gp120 interface in the N49P9.6-FR-BG505 SOSIP.664 complex is 2,441 Å^2^ (1,184 Å^2^ for gp120 and 1,257 Å^2^ for Fab).

Although, as described earlier, the primary N49P9.6-FR-gp120 interface is largely identical in the N49P9.3-FR-gp120 monomer and N49P9.6-FR Fab-SOSIP trimer complexes, the N49P9.6-FR Fab establishes a new set of interactions with the adjacent gp120 protomer (Figures 5B and 5C). These contacts are mediated by the -FR insertion in N49P9.6-FR and are similar, though not identical, to the network of interactions previously reported to promote greater potency in other CD4bs bnAbs including VRC03^30,32^ (Figure 5B). We were able to fully resolve the framework insertion in the N49P9.3-FR Fab-gp120_93TH057_core_e_ complex, thanks to the higher resolution of the data, which helped in our cryo-EM reconstruction. Overall, the framework insertion in N49P9.6-FR seems to better mirror VRC03 than the same insertion when engrafted onto N6-FR or VRC01-FR (Figure S7A). Total BSA for the adjacent protomer is 388 Å^2^ for N49P9.6-FR (194 Å^2^ for gp120 and 194 Å^2^ for Fab) versus 376 Å^2^ for VRC03 (179 Å^2^ for gp120 and 197 Å^2^ for Fab), 298 Å^2^ for N6-FR (138 Å^2^ for gp120 and 160 Å^2^ for Fab), and 385 Å^2^ for VRC01-FR (180 Å^2^ for gp120 and 205 Å^2^ for Fab). N49P9.6-FR primarily makes one hydrogen bond to gp120 Tyr^318^, weak salt bridges with Lys^207^, Arg^304^, and Arg^308^, and van der Waals contacts to Pro^206^ on the adjacent protomer. VRC03 makes similar contacts to the adjacent protomer minus contacts to Arg^304^ and Arg^308^. N6-FR shifts its adjacent protomer contacts more toward Tyr^318^ while VRC01-FR shifts more toward Lys^207^ and Arg^304^ (Figure S7A). N49P9.6-FR’s opening of the trimer is also intermediate between VRC03’s, the most open, and N6-FR and VRC01-FR, the most closed (Table S7). These additional adjacent protomer contacts add approximately 16% more BSA and contribute significantly to N49P9.6-FR’s affinity for the Env trimer.

The FR is not the only method by which CD4bs antibodies have expanded their epitope footprint onto the adjacent protomer. Wild-type VRC01 also contacts the adjacent protomer, but only weakly to one residue, Glu^64^ (Figure S7B). N49P6 from the N49 P7 lineage makes more extensive contacts to the adjacent protomer by stabilizing a closed conformation of the trimer and contacting residues Tyr^61^, Glu^64^, Lys^65^, and His^66^.^53^ 3BNC60 and 3BNC117 utilize a similar but shorter framework 3 insertion as VRC03 to contact adjacent gp120 residues Lys^207^, Ser^306^, Ile^307^, Arg^308^, Gln^315^, and Ala^316^.^55,56^ The IGHV1–2 encoding CD4bs antibody CH31 uses a CDR H1 insertion and framework 1 residues to contact adjacent residues His^66^, Gln^114^, Pro^118^, Pro^206^, Arg^304^, Ser^306^, Ala^316^, and Tyr^318^, and the IGHV1–46 encoding CD4bs antibody 1–18 uses CDR H1 and framework 1 residues to contact adjacent residues Lys^207^, Arg^304^, Ser^306^, Ile^307^, Arg^308^, Ala^316^, and Tyr^318^.^57,58^ The adjacent gp120 contact residues are generally highly conserved among HIV strains, which augment the neutralization breadth of these antibodies (Figure S7B). A more detailed discussion of the nature of these adjacent contacts can be found in the study by Tolbert et al.^53^ The FR insertion has been one of the most translatable methods of configuring adjacent protomer contacts to IGHV1–2 encoding CD4bs antibodies, but the success of this transplant varies.^30–32^ Prior to N49P9.6-FR, the most successful transplant has probably been VRC01.23LS, VRC01-FR with an N-terminal 3 amino acid deletion in the VRC01 light chain, and a substitution of Gly^54^ to Trp in the heavy chain to make it more VRC03-like, highlighting the importance of the primary protomer interface to the success of the framework insertion.^31^

Finally, the cryo-EM reconstruction of the N49P9.6-FR Fab-BG505 SOSIP.664 complex also included the PGT121 Fab, which was utilized as a chaperone in structural studies. Previous trimer structures involving the BG505 SOSIP trimer have only featured an inferred germline version of PGT121, namely the 3H+109L variant, but have never included the fully mature PGT121 antibody itself.^50^ Given this, we decided to investigate how the PGT121 Fab interacts with the Env antigen, to gain deeper insight into its structural and functional implications. As shown in Figure S8, gp120 contact residues are largely identical between PGT121 and 3H+109L PGT121, but the interactions in PGT121 are more protein dependent, which can be seen from the BSA. The total BSA for 3H+109L PGT121 is 2,275 Å^2^ (1,211 Å^2^ from gp120 and 1,064 Å^2^ from Fab), and the total BSA for PGT121 is 2,455 Å^2^ (1,236 Å^2^ from gp120 and 1,219 Å^2^ from Fab). 1,126 Å^2^ of the total BSA is due to protein residues for 3H+109L PGT121, while 1,308 Å^2^ of the total BSA is due to protein residues for PGT121. Contributions from glycan are roughly comparable for both, 1,126 Å^2^ for 3H+109L PGT121 and 1,147 Å^2^ for PGT121. Thus, one way PGT121 seems to have increased its affinity to Env during maturation is to have maximized its interactions with protein residues while maintaining similar interactions with key glycans. Most of these changes are subtle however and can only be seen in aggregate (Figures S8B and S8C).

### Secondary antibody engineering to increase potency

Further engineering of N49P9.6-FR-LS to further improve neutralizing activity was attempted based in part on previous successful structural design efforts applied to bnAbs,^59,60^ as well as attempts to improve the FR contacts to the opposing protomer. Modifications were analyzed using the Therapeutic Antibody Profiler tool,^61^ to assess antibody developability using structure-based metrics, and Rosetta,^62^ to calculate antibody stability or binding energy changes (ΔΔGs) for individual substitutions. Potentially favorable substitutions identified by one or both of those methods were prioritized for testing. In addition, we considered (1) the removal of a possible N-glycosylation motif in the heavy-chain V domain (S60A) given that such a modification was previously shown to be beneficial for antibodies^63^; (2) a CDRL2 loop residue swap (FDDDK49YSGST) from the related VRC01 antibody^59^; and (3) two substitutions in the heavy-chain framework-3 loop based on computational scanning mutagenesis. In total, 11 N49P9.6-FR designs were selected for characterization (Table S8).

All new designs were expressed by transient transfection (see Method details) and assessed for neutralizing activity (breadth and IC50, IC80 potencies) using a screening mini-panel of 21 pseudoviruses (extracted from the larger 119 global panel) comprising viruses that are moderately or completely resistant to the parental N49P9.6-FR-LS. None of the changes led to a statistically significant improvement in the IC50 or IC80 (Figure S9). Interestingly, certain modifications led to improved potency against specific viruses in the panel (i.e., >5-fold improvement in IC50/IC80), including the N-glycan site removal substitution, variable-constant domain hinge substitutions (heavy chain V110T and light chain T108Q), and a structure-based point substitution (light chain T70D). However, overall improvements in median potency were not significant when the modified constructs were tested on the larger 119 pseudovirus panel (not shown). Interestingly, some designs, particularly the two “YSGST” CDRL2 swap-containing variants, which included CDRL2 residues from another VRC01-class antibody (VRC01), showed substantially reduced breadth and potency versus N49P9.6-FR. Overall, the secondary engineering effort yielded no further improvements; thus, we proceeded with the original design of N49P9.6-FR-LS.

### Tertiary antibody engineering for developability

In our final engineering stage, we attempted to identify modifications that could potentially improve the scalability and manufacturability of N49P9.6-FR-LS under cGMP production, without loss of neutralizing activity or other desirable properties. Given the aforementioned PK data, which demonstrably increase circulating half-life *in vivo* (Table 1), all variants expressed a heavy-chain sequence containing the “LS” mutation.

The first round of optimization was performed in a phage library panning format based on efficient HIV CH505 gp160 binding. Sixty-two candidates were identified during phage-Fab panning that bound at a 0.8 nM target concentration after either temperature or chemical challenge (see Methods). These variants were produced in a transient expression system and tested for retention of neutralization breadth and potency versus the parental constructs. Detrimental mutations were defined as ones with >5-fold lower BaL EC50 gp120 binding on ELISA and/or ≥5-fold increase in IC50 or IC80 against any 1 virus in the 10 pseudovirus panel (extracted from the larger 119 global panel). Eleven candidates demonstrated unacceptable loss of neutralization; one of these also had >5-fold decrease in binding by EC50.

Of the remaining 51 variants with fully preserved breadth and acceptable potency, 5 showed 2- to 4-fold median improvements in IC50 and IC80. The 51 variants were then further screened (see Methods) to assess conformational and colloidal stability characteristics (Figure S10) favoring manufacturability properties.^64–66^ The variants were ranked according to each of the parameters and the variant with the most improved measure in each category (without worsening in another category) was chosen for further screening.

This exercise identified 6 variants exhibiting improved conformational and/or colloidal stability. These variants were then combined with the 5 that had demonstrated more potent neutralization for further screening. However, there were two variants that were present in both 6 variant and 5 variant sets that demonstrated both conformational/colloidal and neutralization improvement, meaning a total of 9 variants underwent further screening. Stable pools were made from these 9 variants and re-tested for neutralizing activity against 60 pseudovirus panel (one-half of the 119 Global Tier 1–3 panel) and conformational/colloidal properties. The neutralization IC50 and IC80 were all within 2-fold of the parental N49P9.6-FR-LS strain, while the only measures with improvement seen between the transient transfection and stable pool material were in PEG solubility and thermal hold in one variant (M-5817) and PEG solubility and T1 in a second variant (M-5809) (data not shown). Thus, the apparent neutralization gains seen with the 10 virus panel were not reflected in a larger panel.

Using a Residual Artificial Neural network for the Design of Antibodies (RANDŸ), we identified a 3 amino acid deletion in N terminus of the light chain as a biophysical liability. This is a feature common to anti-CD4bs bNAbs.^26,31^ In the N49 P series, mutations in the leader sequence cause the truncation of an “ASA” sequence (as determined by N-terminal sequencing and peptide mass spectrometry; data not shown) via post-translational processing. Accordingly, one optimization strategy was to restore this N-terminal tripeptide sequence in the light chain using a leader sequence resistant to proteolysis. Variants containing this modification were given the germline or “_GL” designation. In the final phase of development, wild-type and “_GL” forms of M5809 and M-5917, as well as parental N49P9.6-FR-LS, were each made.

These six variants underwent another round of neutralization testing (119 global panel and a 100 clade C virus panel). These analyses again showed IC50 and IC80 that were comparable among all variants and the parental bNAb (<2-fold differences), although one was statistically significant (Figure S11). These six variants underwent another round of biophysical testing, and based on improvements in thermostability, PEG solubility, and viscosity compared to the parental form N49P9.6-FR-LS, variant M-5817_GL demonstrated the broadest improvement overall (Table S9). Thermal stability of M-5817_GL was improved as evinced by the additional melting transition at 70°C, which corresponded to a reduction in precipitation upon thermal hold (Table S9). While the conformational stability was improved in the thermal stress methods, a slight reduction in the inflection point during chemical unfolding was observed (Table S9). Viscosity curves (Figure S12) were generated for parental N49P9.6-FR-LS and M-5817_GL to validate that improvements in colloidal stability were also evident under GMP-relevant concentrations and formulation conditions (Table S9). As indicated by the improved colloidal stability measured by biophysical analysis, the optimized candidate showed a significant reduction in viscosity when formulated at high concentrations. M-5817_GL (henceforth referred to as eN49P9) was the final candidate chosen for further clinical development.

## DISCUSSION

Clinical experience has shown that individual bnAbs suffer the same shortcomings as most single-agent HIV countermeasures, specifically, escape due to virus genomic diversity and mutability. One mitigation approach is to combine bnAbs from different specificity classes, each with nonoverlapping gaps in variant coverage.^37,39,67^ A complimentary approach is to engineer rational improvements into near pan-neutralizing bnAbs. It stands to reason that combinations of such antibodies would provide the strongest prospects for clinical efficacy.

Rational engineering of bnAbs must strike a balance between increasing breadth and potency while preserving stability, acceptable *in vivo* PK, and, importantly, manufacturability under cGMP. Improvements in one property can lead to deterioration in another. The CD4bs bnAbs are particularly challenging as their activity depends on distinct Fab architectures that facilitate main-chain and side-chain Env interactions.^26,52,68^ Such features arise from high rates of somatic hypermutation,^26,52,69^ framework mutations, and characteristic deletions.^31,70^ Thus, recognized techniques for increasing antibody potency may not apply, e.g., a 54W/F/Y substitution in the heavy chain can drive anti-CD4bs bnAbs to polyreactivity.^31^ Recognizing these hurdles, we pursued a stepwise, comprehensive approach toward the engineering of the enhanced bnAb, eN49P9, with an aggregate profile supporting clinical applications.

Our starting point was N49P9.3, which among other “N49P family” members demonstrated superior breadth and potency, unique contacts with gp120 (Figure S4), and other features distinct from the canonical VRC01 types of anti-CD4bs bnAbs.^26^ N49P9.3 was engineered to N49P9.6 by reverting the 1^st^ position of the constant regions in the heavy and light chains to germline and engrafting the framework-3 loop from VRC03 to enable quaternary interaction with a second gp120 protomer. Notably, this latter modification led to gains in neutralizing potency greater than seen with N49P7-FR or VRC01.23LS modified in a similar manner.^31^ The structural basis (Figure 5) for the superior performance of N49P9.6-FR likely stems from the orientation of VRC03 framework-3 insertion, which closely resembles VRC03 itself^32^ and allows more extensive protomer contacts with adjacent gp120 protomers versus what occurs with similarly engineered N6-FR or VRC01-FR. Such features could increase binding avidity and consequently neutralization breadth and potency.

It was encouraging that N49P9.6-FR engineering also boosted ADCC against infected cells expressing recently recovered Env variants (Figure S3). However, the LS mutation, which improved PK (Table 1), appeared to further modulate this activity, lowering or increasing ADCC scores, depending on the variant. The basis for such modulation merits further analyses at the structural level. The involvement of ADCC in N49P9.6-FR-LS efficacy was further suggested by the *in vivo* experiments (Figure 4) showing that bnAb potency was substantially greater in the CD34-NSG-IL15 mice versus the CD34-NSG-SGM3 mice challenged with the same virus. Although there were differences between the strains in relevant cell subsets (e.g., % CD4, CD4/CD8 rations), the variances were on average only 2- to 3-fold. However, % CD45^+^, CD56^+^ human NK cells were >10-fold higher in the cd34-NSG-IL15 strain versus levels in the CD34-NSG-SGM3 animals (which were very low to undetectable) in agreement with previous literature.^47,71–73^ We interpret the aggregate data to indicate that direct neutralization is the predominant mechanism of protection in CD34-NSG-SGM3 mice, whereas Fc-mediated effector mechanisms (such as ADCC by NK) can be recruited in the CD34-NSG-IL15 mice, thus boosting N49P9.6-FR efficacy. This scenario is consistent with previous demonstrations that NK recruitment contributes to the efficacy of PGT121 in mice expressing human IL-15.^17^

N49P9.6-FR engineering also provided advantages in bnAb class combinations, which will almost certainly be needed to provide clinical efficacy and escape resistance. Multi-parameter neutralization measures *in vitro* were uniformly superior when N49P9.6-FR-LS was used alone or in combination as the anti-CD4bs class component (Figure 3), outperforming analogous combinations with all other CD4bs bNAbs. These data agree with our previous findings that anti-CD4bs and anti-V3 bnAbs can bind to the same particle.^74^ Such redundancy is expected to mitigate escape risk and facilitate cooperative or synergistic bnAb action.^36,37,75^ Notably, N49P9.6-FR + PGDM1400 + PGT121 exceeded IIP >5log10 against more than 70% of test viruses and was predicted to cover >80% of test viruses with ≥2 bnAbs active.

Surprisingly, structure-guided engineering failed to improve the overall neutralization profile of N49P9.6-FR-LS. While certain modifications led to more potent neutralization of a few variants, they reduced potency against others (Figure S8). One limitation of our modeling approach was that available SOSIP trimers represent variants already sensitive to the bnAb and as such may fail to detail the structural basis for resistance. In addition, SOSIP trimers are static structures lacking thermodynamic features impacting bnAb binding and function. It was recently shown that anti-CD4bs bnAbs variably favor open versus closed or intermediate Env transition states^76^; extreme breadth can stem from non-selective binding across this structural landscape. Thus, success in future bnAb engineering efforts will likely require modeling tools encompassing both the structures and thermodynamic properties of resistant Env variants.

Our efforts to engineer manufacturability while retaining neutralization potency and breadth was more productive, yielding one variant (eN49P9) with 6 heavy chain and 3 light-chain mutations, as well as an uncleaved light-chain N terminus. This variant has improved PEG solubility and thermostability and retains other desirable biophysical characteristics. Moreover, the eN49P9 variant had less than a 2-fold change in neutralization potency in the 119 multi-clade compared to parental N49P9.6-FR-LS. Overall, our findings suggest that a near-pan neutralizing bnAb against the CD4bs can be engineered for both enhanced antiviral activities and manufacturability. New antibodies such as eN49P9 may be considered for future clinical testing in combination with enhanced bnAbs of other specificity classes.

## RESOURCE AVAILABILITY

### Lead contact

Requests for further information and resources should be directed to and will be fulfilled by the lead contact, Mohammad M. Sajadi (msajadi@ihv.umaryland.edu).

### Materials availability

All unique/stable reagents generated in this study are available from the lead contact without restriction.

### Data and code availability

- The atomic coordinates generated in this study are publicly available as of the date of publication at the RCSB (https://www.rcsb.org) with the following PDB accession codes: PDB: 7UOJ (PGT121 and N49P9.6-FR-LS), PDB: 6OZ3 (N49P9.1), PDB: 7SX6 (N49P9.3), PDB: 7SX7 (N49P9.3-FR). The EMDB accession code is EMD-26648 and they are publicly available as of the date of publication. The nucleotide data of mAbs N49P9, N49P9.3, and PGT121 are publicly available at GenBank (https://www.ncbi.nlm.nih.gov/genbank), and their accession codes are listed in the key resources table.
- This paper does not report original code.
- Any additional information required to reanalyze the data reported in this paper is available from the lead contact upon request.

## STAR★METHODS

### EXPERIMENTAL MODEL AND STUDY PARTICIPANT DETAILS

#### Regulatory approval for mouse studies

All mouse studies approved by the University of Maryland School of Medicine, Baltimore, IACUC, and performed in compliance with federal regulations, policies, and guidelines, including the Animal Welfare Act and the Public Health Service Policy. Mice are housed in microinsulator cages in Specific Pathogen Free (SPF) rooms.

#### FcRn−/− hFcRn transgenic mice

PK testing was carried out in mice from strain B6.Cg-Fcgrttm1Dcr Prkdcscid Tg(FCGRT)32Dcr/DcrJ (abbreviated as *scid* FcRn−/− hFcRn Tg) (Strain # 018441; Jackson Laboratories, Bar Harbor, Maine). These mice, which harbor a knockout allele of the FcRn α-chain (Fcgrt^tm1Dcr^) and express the human FcRn α-chain, have been extensively studied and used for measuring IgG half-life of human mAbs and Fc engineered mutants. Mice (9 weeks old, equal male and female) were used in this study.

#### NSG-SGM3 mice

NSG-SGM3 (NOD.Cg-*Prkdc*^*scid*^ *Il2rg*^*tm1Wjl*^ Tg(CMV-IL3,CSF2,KITLG)1Eav/MloySzJ) were from Jackson Laboratories (Strain #013062). Newborn mice, (equal male and female), were humanized with human CD34^+^ stem cells as detailed below. Assignment in experimental groups was done randomly, and after successful humanization.

#### NSG-IL15 mice

NSG-IL15 (NOD.Cg-*Prkdc*^*scid*^ *Il2rg*^*tm1Wjl*^ Tg(IL15)1Sz/SzJ) were from Jackson Laboratories (Strain #030890). Newborn mice, (equal male and female), were humanized with human CD34^+^ stem cells as detailed below. Assignment in experimental groups was done randomly, and after successful humanization.

#### Cell lines

TZM-bl cells (sex: female) were obtained from the NIH AIDS Reagent Program (Bethesda, MD) and cultured in 5% C0_2_ at 37°C in RPMI medium from Gibco (Gaithersburg, MD) supplemented with 10% heat inactivated FBS with 100 units/mL of penicillin (Gibco) and 100 μg/ml of streptomycin (Gibco). FreeStyle 293-F Cells (sex: female) were obtained from Thermo Fisher Scientific (Waltham, MA) and were cultured in 5% C0_2_ at 37°C in FreeStyle 293 Expression Medium from Gibco. CEM.NKR_CCR5_ cells were kindly provided by Dr. John Kappes, and cultured in and cultured in 5% C0_2_ at 37°C in RPMI medium (Gibco) supplemented with 10% heat inactivated FBS (Nucleus Biologics, San Diego, CA) with 100 units/mL of penicillin (Gibco) and 100 μg/ml of streptomycin (Gibco). HD-BIOP3 GS Null CHO K1 (sex: female) was obtained from Revvity (Waltham, MA; formerly known as Horizon Discovery Limited) cultured in CD OptiCHO growth medium (Gibco) containing 4 mM glutamine at 5% C0_2_ at 37°C. Just Evotec has access, through non-exclusive in-licensure, to Horizon Cell Line Technology, i.e., Horizon’s proprietary HD-BIOP3 GS Null CHO K1 cell line.

### METHOD DETAILS

#### Antibody isolation and production

The B-cell database of volunteer N49, previously assembled^26^, was searched for all clones with characteristic CD4bs deletion in the complementarity-determining region (CDR)L3, and found sequences aligned to identify those in the N49P9 family. The identified VH or VL region clones were cloned into an expression vector upstream to a human IgG1*03 backbone for heavy chain and either a k or λ light chain expression vector. The paired plasmids were used to transiently transfect 293 Freestyle cells. Transfectant supernatants were used for purification of the mAbs using protein A affinity chromatography.

#### ELISA

The HIV binding ELISA was performed with monomeric BaL-gp120 antigen. BaL-gp120 was directly coated (4 μg/ml) on the plates, but otherwise ELISA was the same as previously described.^26^ Each assay performed in duplicate at multiple dilutions and EC50 calculated using GraphPad Prism 10 software (Boston, MA).

#### Neutralization assay

HIV-1 neutralization testing was performed using a luciferase-based assay in TZM.bl cells, as previously described.^92^ Briefly, three- or five-fold serial dilution of plasma, supernatant, or monoclonal antibodies were tested against a panel of Env-pseudotyped viruses in duplicate. Following a 48 hour incubation, the reduction in luciferase expression following a single round of virus infection was measured using Bright-Glo luciferase reagent and GloMax luminometer (Promega, Madison, WI). The concentrations of antibody that inhibited 50% or 80% of viral infection (IC50 and IC80 titers, respectively) were calculated using 5-parameter curve fitting (LabKey NAb Tool).^93^ (Figures S2, S9, S11, Tables S1–S3, and S8).

#### Clinical polyreactivity testing

Antibodies were tested in a Clinical Laboratory Improvement Amendments (CLIA)-certified clinical lab (University of Maryland Medical Center) for anti-nuclear (ANA), anti-centromere, anti-Jo1, anti-Sjögren’s-syndrome-related antigen A (SSA-52), anti-Sjögren’s-syndrome-related antigen A (SSA-60), anti-Sjögren’s-syndrome-related antigen B (SSB), anti-Smith (ENA), and anti-smith/RNP (ENA) antibodies. For all assays, mAbs were tested at 25 μg/ml, with the positive and negative controls that were assay-specific (Table S4).

#### ADCC assay

##### Env-IMC construction and virus production

To construct Env-IMCs which contain the ectodomain of the Envelope of interest (HxB2 positions 41–687) and an isogenic NL4.3 backbone as previously described,^94^ partial *envelope* genes were amplified by PCR to produce amplicons which contained a 20 base pair overlap with the pNL4.3 backbone on both the 5’ (HxB2 positions 6353–6335) and 3’ (HxB2 positions 8283–8299) ends. The pNL4.3 plasmid (BEI Resources, Cat. no. ARP-114) was then PCR amplified to generate a product which excluded the Env ectodomain nucleotides but included the signal peptide and endodomain regions of NL4.3 *envelope*. The pNL4.3 and partial *envelope* PCR products were then fused using NEBuilder HiFi DNA Assembly (NEB, Ipswich MA) per the manufacturer’s recommendations to produce an NL4.3-*env*.ecto chimeric HIV infectious molecular clone (Env-IMC) which contained a complete, in-frame *envelope*. Viruses were produced by transfecting the Env-IMCs into HEK293T cells using Dreamfect Gold transfection reagent (OZ Biosciences, San Diego CA). Virus-containing supernatants were harvested 48 h following transfection, clarified by 0.45 μm filtration and adjusted to 10% FCS (Nucleus Biologics).

##### Target cell infection

1×10^6^ D660 cells (CEM.NKR_CCR5_ cells which contain GFP and Renilla *luciferase* genes under the control of the HIV-1 LTR; kindly provided by Dr. John Kappes, University of Alabama at Birmingham) were infected with 1mL of virus. Briefly, 2μL of Lentiblast (OZ Biosciences) was added to 1mL of virus and incubated for 10 minutes. D660 cells were then pelleted by centrifugation at 485xg for 5 minutes and the virus/Lentiblast mixture was added to the cells. Cells and virus were centrifuged at 1200×g for 2 hours at 30°C. After centrifugation, cells were resuspended in RPMI10 to a final concentration of 5×10^5^ cells/mL and incubated for 72 hours.

##### Renilla luciferase-based antibody-dependent cell-mediated cytotoxicity (ADCC) assay

A modified version of the LucR-based ADCC assay previously described^95^ was conducted: the day prior to the ADCC assay, cryopreserved peripheral blood mononuclear cells (PBMCs) to be used as effectors in the assay were thawed in R10, counted and assessed for viability and resuspended in R10 overnight. On the day of the assay, infected D660 cells were counted, assessed for viability (viability was ≥80% to be used in the assay) and the concentration was adjusted to 2 × 10^5^ viable cells/mL (5 × 10^3^ cells/well). PBMCs were then counted, assessed for viability, pelleted and resuspended in the infected D660 cells at a concentration of 6 × 10^6^ PBMCs/mL (1.5 × 10^5^ PBMCs/well; effector: target cell ratio of 30:1). Heat-inactivated autologous plasma was serially diluted. The effector/target cell mix and antibody dilutions were plated in opaque 96-well half-area plates, centrifuged at 300 × g for 1 minute after 30 minutes incubation at room temperature, and then incubated for 5.5 hours at 37°C, 5.5% CO_2_ to allow ADCC-mediated cell lysis to proceed. After 5.5 hours, ViviRen substrate (Promega) was diluted 1:500 in R10 and added 1:1 to the assay wells. The substrate generates luminescence only in live, infected cells; not in dead or lysed cells. The final readout was the luminescence intensity generated by the presence of residual intact target cells that have not been lysed by the effector population in the presence of ADCC-mediating antibodies. The percentage of specific killing was calculated using the formula

$$\%\text{specific}\;\text{killing}=\frac{\text{RLU}\;\text{of}\;\text{target}+\text{effector}\;\text{well}-\text{RLU}\;\text{of}\;\text{test}\;\text{well}}{\text{RLU}\;\text{of}\;\text{target}+\text{effector}\;\text{well}}\times 100$$

In the analysis, the RLU of the target + effector wells represent lysis by natural killer (NK) cells in the absence of any source of antibody. The specific killing activities in the ADCC assay were summarized for each mAb and virus by computing the area under the dilution curve (AUC) as the mean of the specific killing activities across the dilution series. Specific killing activities less than 10% were assigned a value of 0 prior to AUC calculation (Figure S3).

#### Antibody combination analysis

##### BnAb and bnAb combination modeling

To compare individual bnAbs, we predicted inhibitory concentration (IC)99 and IIP for each bnAb versus each virus using the mathematical modeling previously outlined.^36^ Briefly, neutralization curve slopes were calculated using IC50 and IC80 titers and these with IC50 titers characterize the Hill curve that determines the full neutralization curve, which then allows calculation of IC99 (concentration at which 99% of the viral sample is neutralized) and IIP (Log10 fold reduction in a single round of replication in the presence of bnAb). For bnAb combinations the Bliss-Hill model^36^ was used that can predict the full neutralization for the bnAb combination using individual bnAb IC50 and IC80 titers. This mathematical description of the full curve can be solved to obtain IC50, IC80 and IC99 titers and IIP for the combination. Titers for bnAb combinations are calculated assuming each bnAb in the combination is present at the same concentration, and they are reported as the sum of concentrations of each bnAb (e.g. if a 2-bnAb combination has an IC50 = 1 μg/ml, then each bnAb is present at 0.5 μg/ml; similarly if a 3-bnAb combination has IC50=1 μg/ml, then each bnAb is present at 0.33 μg/ml). For ranking, 6 summary metrics were used for individual bnAbs: 1) IC80 geometric mean, 2) IC80 < 1 μg/ml breadth, 3) IC99 geometric mean, 4) IC99 < 10 μg/ml breadth, 5) median IIP, 6) IIP>5Log10 breadth. For bnAb combinations, additionally IC80 breadth with 2 or more bnAbs active was also considered. Each individual bnAb or bnAb combination was compared to that of other individual bnAbs or combinations with the same number of bnAbs. For each metric, rank was calculated as 1 + the number of bnAb/combinations strictly better (not better than or equal to) than the bnAb or combination in question.

#### Antibody engineering for potency

In order to enhance binding affinity and avidity, we first undertook a strategy to engrafting the heavy chain framework 3 loop (“FR”) from VRC03 to enable quaternary interaction with a neighboring gp120 protomer, as previously reported for other antibodies.^30,32^ Thus, N49P9.6 was engineered into N49P9.6-FR, each Fab arm with an unconventional bnAb architecture that presents two paratopes reactive with two neighboring protomers on the Env trimer, mimicking the quaternary binding mode of CD4.^33^ To optimize the N49P9.6-FR antibody, we utilized multiple rational and structure-based design approaches to improve its biophysical properties and antigen targeting. Based in part on previous successful HIV antibody design efforts,^59,60^ we assessed germline revertant mutations and consensus residue mutations from VRC01-class antibodies using the Therapeutic Antibody Profiler (TAP) tool,^61^ which assesses antibody developability using structure-based metrics, as well as Rosetta,^62^ which was used to calculate antibody stability or binding energy changes (ΔΔGs) for individual substitutions based on modeling. Favorable substitutions based on one or both of those methods were prioritized for experimental testing. We additionally tested a substitution to remove a possible N-glycosylation site in the heavy chain V domain, given that N-glycan removal was previously shown to be effective for antibodies,^63^ as well as a CDRL2 loop residue swap from the related VRC07 antibody.^59^ Finally, two substitutions were selected based on computational scanning mutagenesis of the heavy chain framework-3 loop in Rosetta and favorable predicted Env targeting and/or thermostability.

#### Antibody engineering for developability

Sequence stability violations were determined for N49P9.6-FR using a Just proprietary Residual Artificial Neural network for the Design of Antibodies (RANDŸ). VH and VL were designed based on all possible amino acid combinations at the identified sites. These VH and VL designs were DNA synthesized at Twist Bioscience (San Francisco, CA) as library pools for the VH and VL regions, separately. The V-regions were cloned into a phagemid vector and M13 Fab display libraries were generated as described.^96^

The gp160 target (CH505TFchim.6R.SOSIP.664v4.1) was randomly biotinylated on primary amines using the EZ-link NHS-Biotin kit per manufacturer’s recommendations (ThermoFisher Scientific, Waltham, MA). Multiple rounds of Fab phage panning were performed using a MagBead Kingfisher platform. The first panning round used 100nM biotinylated gp160 in phosphate buffered saline (PBS) to allow Fab-phage binding. Four, 10min PBS washes were performed followed by phage elution at pH 2.5 via 0.2M glycine. Subsequent rounds had multiple arms where temperature challenge was conducted in one arm whereas GnHCl challenges were conducted in the other. Temperatures ranged from 65°C to 75°C and GnHCl concentrations ranged from 0.25 to 1.0 M to increase selection of more stable Fabs. Each library challenge was for 10min. Additionally, the gp160 target concentration was decreased from 20 nM in the second round to 0.8nM in the last round. Sanger sequencing and polyclonal ELISA using the same gp160 as used in the panning was performed after rounds 1–3 to confirm Fab enrichment. 48–96 Fabs from all arms were converted to IgG1 in a batch, two step, high-throughput cloning process. These sequenced plasmids were transiently transfected in duplicate into HEK293 cells for small-scale expression in 96well blocks as per manufacturer’s recommendations (ThermoFisher Scientific, Waltham MA). After 4 days of expression, the supernatants were harvested and antibody secretory titers measured via BLI. These crude supernatants were then assessed for neutralization activity in up to 10 pseudovirus panel (first tested against 5 pseudoviruses, and if no loss of neutralization, tested against additional 5 pseudoviruses). Loss of neutralization defined as > 5 fold loss of IC50 or IC80 against any single pseudovirus. The top candidates as determined by preservation of neutralization activity were scaled-up in the 293 transient expression system as described but rather in 24well blocks or 125mL flasks to provide enough material for further downstream analytics. The supernatants were harvested after a 4-day expression and the titers measured via BLI. The secreted antibodies were Protein A purified. Samples were buffer exchanged against 10 diavolumes of 20mM sodium phosphate, 150mM sodium chloride, Ph 7.1 (PBS) using a centrifugal filter with a 30 kDa molecular weight cut off (Amicon). After buffer exchange, samples were normalized to 1 mg/ml using a Lunatic protein concentration plate format instrument (Unchained Labs, Pleasanton, CA). The antibodies were then advanced for biophysical characterization and further neutralization activity assessment (Figure S1).

##### Generation of stable pool for final candidate variants

HD-BIOP3 GS Null CHO K1 cells were cultured in CD OptiCHO growth medium containing 4 mM glutamine. One day prior to transfection, host cells were passaged at 1E6 cells/mL to maintain the cells in exponential growth phase. HT method transfection was performed to create stable pool replicates, each receiving a total of 10ug of N49P9.6-FR expression vector and transposase mRNA using ECM-830 BTX electroporator. Immediately after transfection, cells were transferred to a 24 DWP containing 2 mL of warm glutamine supplemented CD OptiCHO for a 3-day recovery. Selection of transfectants began with culturing the cells in glutamine-free CD OptiCHO medium. Stable pools reached 98% viability by selection day 11 and were subsequently subjected to a 10-day fed batch production for early material generation, evaluation of productivity and protein quality prior to single-cell cloning.

##### Differential scanning fluorimetry (DSF)

Thermal transition temperature(s) and weighted shoulder scores were determined by DSF according to the method previously described.^97^ A single biological sample was divided, and the assay ran twice per molecule type. Additional information was also obtained from a parameter termed the weighted shoulder score (WSS) which accounts for multiple pieces of information from the unfolding curve^98^ (Table S9).

##### Low pH stability

Stability during a low pH hold was determined according to the method previously described.^97^ The increase in high molecular weight of the low pH exposed sample as compared with the control sample was reported. Any increase above 10% high molecular weight after low pH is considered undesirable and will present additional manufacturing challenges (Figure S10).

##### Chemical unfolding

The chemical unfolding assay was completed as previously described,^99^ with some modifications. After a 3-day incubation in 32 guanidine hydrochloride (GND) concentrations, the samples were measured on a Fluorescence Innovations SUPR-UV plate reader (excitation: 275 nm, emission: 300–440 nm). The measured fluorescence intensity at 362 nm was corrected for scattering and stray light, the unfolding curve was generated by graphing each corrected intensity against the GND concentration and the inflection point was reported. Inflection point range between 1.5 to 2.5 molar GND has been observed internally, with higher inflection points being reflective of higher conformational stability (Figure S10).

##### Relative solubility

Solubility was assessed according to the method previously described.^100^ Analysis was done in PBS buffer (20mM sodium phosphate and 150mM sodium chloride pH 7.1) and a final PEG 10,000 concentration ranging from 7.2% to 9.6%. Percent recovery relative to a 0% PEG control was determined and average recovery across the PEG concentration range was reported.

##### Self-interaction nanoparticle spectroscopy (SINS)

SINS measurements were performed according to the method previously described.^101^ Briefly, gold nanoparticles (Ted Pella, Redding, CA) were conjugated overnight with an 80:20 ratio of anti-human and anti-goat antibodies (Jackson Immuno Research, West Grove, PA). Unreacted sites were blocked using an aqueous 0.01% (w/v) polysorbate 80 solution. Conjugated gold nanoparticles were then concentrated by centrifugation and removal of 95% of the supernatant. Analysis was carried out in PBS (20mM phosphate, 150mM NaCl, pH 7.1) at a protein concentration of 0.05 mg/ml reacted with 5 μl of concentrated conjugated gold nanoparticles. After a 2-hour incubation, absorbance spectrum from 400–600 nm was collected using a Spectrostar Nano plate reader at 2nm steps. The wavelength maximum of the spectrum peak is reported. Three assay replicates were made from each of the pooled expressed antibodies.

##### Standup monolayer absorption chromatography (SMAC)

SMAC measurements were performed according to the method previously described.^102^ Retention times were determined using a Dionex UPLC equipped with a Zenix column (Sepax Technologies) and a running buffer comprised of 150mM sodium phosphate pH 7.0.

##### Polyreactivity

Polyreactivity testing was performed according to the method previously described.^103^ An ELISA assay was used to test against 4 different antigens: KLH, Insulin, dsDNA and Cardiolipin. Samples are diluted to 1 μg/mL and a secondary anti-human antibody is used to detect the amount of protein that has bound to the different antigens. After substrate addition, absorbance is measured at 405 nm and subtracted against a PBS blank sample. An absorbance range between 0 to 3 has been observed (Table S4).

##### Viscosity

Samples were buffer exchanged into 10mM acetate, 9% sucrose, pH 5.2 and prepared at various concentrations from 85 mg/mL to 175mg/mL as measured in triplicate by Solo VPE (Repligen, Waltham Massachusetts). All viscosity measurements were performed at 20°C on an MCR-92 cone and plate rheometer (Anton Paar, Graz Austria) equipped with a CP20–0.5 cone. A shear rate sweep from 50 to 2000 1/s was performed using a sample volume of 25uL per measurement. Viscosity results for sheer rates between 500 and 1000 1/s were averaged and reported per protein concentration (Figure S12).

#### X-ray crystallography

##### Protein expression and purification

BG505 SOSIP.664 and Clade A/E gp120_93TH057_core_e_ were expressed in HEK293 GnTI^−^ cells. Cells were first seeded at 1 × 10^6^ cells/ml (viability >90%) into an expression flask and then transfected with plasmids encoding for the BG505 SOSIP.664 or Clade A/E gp120_93TH057_core_e_ envelope trimer and furin at a molar ratio of 4:1 (SOSIP:furin), as previously described.^83,104^ Transfected cells were incubated for seven days at 37°C, in a humidified atmosphere of 8% CO_2_ on a shaker rotating at 125 rpm. Cell supernatants were then collected, centrifuged and filtered through a 0.22 μm PES membrane to remove cell debris. BG505 SOSIP.664 trimer was purified by N49P9.1 affinity chromatography, i.e. N49P9.1 IgG covalently bound to protein A. BG505 SOSIP.664 was eluted from the column with 3 M magnesium chloride. Eluted protein was immediately diluted in DPBS and then the buffer was exchanged to DPBS using Amicon microcon centrifugal concentrators (Millipore Sigma). Clade A/E gp120_93TH057_core_e_ was purified by 17b affinity chromatography, i.e. 17b IgG covalently linked to protein A. Eluted protein was immediately mixed with 0.1 M Tris-HCl pH 8.5 to raise the pH and the buffer was then exchanged to DPBS using Amicon microcon centrifugal concentrators. Clade A/E gp120_93TH057_core_e_ and BG505 SOSIP.664 was further purified by size exclusion chromatography (SEC) using a Superdex 200 Increase 10/300 GL column (Cytiva) equilibrated with Dulbecco’s phosphate buffered saline (DPBS) buffer. Fabs were produced by papain digestion of IgGs. Briefly IgGs were incubated at 37°C with immobilized papain (Thermo Fisher) for 3–4 hours. Fabs were then separated from Fcs and undigested IgGs by passage over a HiTrap protein A column (Cytiva). Fabs were further purified by gel filtration chromatography on a Superdex 200 16/60 column equilibrated in DPBS. Purified Fabs were then concentrated for use in complex formation.

##### X-Ray complex formation and purification

Clade A/E gp120_93TH057_core_e_ that had been produced in GnTI^−^ cells had its glycans removed with EndoH_f_ (New England Biolabs) before its use in complex formation. Briefly, ten units of EndoH_f_ were added per microgram of protein. The protein was incubated at 37°C overnight and the EndoH_f_ then removed by passage over an amylose column. Complexes were made by mixture of deglycosylated gp120_93TH057_core_e_ with an excess of Fab at a ratio of 1.1:1 (Fab:gp120). Complexes were purified by gel filtration chromatography on a Superdex 200 16/60 column equilibrated with 10 mM Tris-HCl pH 7.2 and 100 mM ammonium acetate. Purified complexes were concentrated to approximately 5–10 mg/ml for use in crystallization trials.

##### Crystallization

Initial crystallization trials were done with commercially available sparse matrix crystallization screens from Molecular Dimensions (Proplex Eco and MacroSol Eco) using the hanging-drop, vapor diffusion method. The screens were incubated at 21°C and monitored periodically for the production of protein crystals. Conditions that produced crystals were then reproduced and optimized. N49P9.1 Fab-gp120_93TH057_core_e_ complex crystals grew from 12% PEG 6000 and 0.1 M Bis-Tris pH 6.5. N49P9.3 Fab-gp120_93TH057_core_e_ complex and N49P9.3-FR Fab-gp120_93TH057_core_e_ complex crystals grew from 10% PEG 4000, 0.1 M 4-(2-hydroxyethyl)-1-piperazine ethanesulfonic acid (HEPES) pH 7.5, and 0.1 M magnesium chloride. Prior to data collection crystals were briefly soaked in crystallization condition supplemented with 20% 2-methyl-2,4-pentanediol (MPD) as a cryoprotectant and then flash frozen in liquid nitrogen (Figure S4).

##### Data collection and refinement

Diffraction data of the N49P9.3 Fab-gp120_93TH057_core_e_ complex was collected at the Stanford Synchrotron Radiation Light Source (SSRL) beam line 9.2 and data for the N49P9.1 Fab-gp120_93TH057_core_e_ and N49P9.3-FR Fab-gp120_93TH057_core_e_ complexes were collected at SSRL beam line 12-2. Both beam lines were equipped with Pilatus area detectors. The N49P9.1 Fab-gp120_93TH057_core_e_ complex crystals belonged to space group I2_1_2_1_2_1_ with cell dimensions of a=104.8 Å, b=110.5 Å, and c=152.2 Å and diffracted to 3.15 Å resolution. N49P9.3 Fab-gp120_93TH057_core_e_ complex crystals belonged to space group P1 with cell dimensions of a=62.2, b=68.2, c=115.1, α=90.3°, β=102.3°, and ɣ=90.3° and diffracted to 3.4 Å resolution. N49P9.3-FR Fab-gp120_93TH057_core_e_ complex crystals belonged to space group P1 with cell dimensions of a=60.4, b=65.4, c=112.4, α=90.0°, β=104.9°, and ɣ=90.0° and diffracted to 2.15 Å resolution. The N49P9.1 complex structure was solved by molecular replacement with Phaser from the CCP4 suite,^91^ based on the coordinates of gp120 from PDB ID 3TGT and the VRC01 Fab from PDB ID 4RFE for the N49P9.1 Fab. The N49P9.3 and N49P9.3-FR complex structures were solved using the refined coordinates from the N49P9.1 complex structure (PDB ID 6OZ3) (Figure S4; Figure S5). Refinement was done with Refmac^86^ and/or Phenix,^87^ coupled with manual refitting and rebuilding using COOT, as described in.^89^ The quality of the final refined models were monitored using the program MolProbity,^90^ as described in.^89^ All illustrations were prepared with the PyMol Molecular Graphic suite (http://pymol.org) (DeLano Scientific, San Carlos, CA, USA). Data collection and refinement statistics are shown in Table 2. Final structures have been deposited in the Protein Data Bank (PDB) with accession codes 6OZ3, 7SX6, and 7SX7. N49 structures were compared to VRC03 (PDB ID 6CDI), VRC01-FR (PDB ID 6NNF), and N6-FR (PDB ID 6NM6).

#### Cryo electron microscopy (CryoEM)

##### CryoEM sample preparation and data collection

Protein complex was made by incubating an excess of PGT121 and N49P9.6-FR Fabs with GnTI^−^ produced BG505 SOSIP.664 trimer overnight at room temperature at a molar ratio of 10:10:1, respectively. The complex was purified by SEC on a Superdex 200 Increase 10/300 GL column (Cytiva) in DPBS buffer. The complex peak was pooled and concentrated to 2.3 mg/ml. 5 μl of protein complex was deposited on a holey copper grid (QUANTIFOIL R1.2/1.3, 200 mesh, EMS cat# Q250-CR1.3) or a carbon film copper grid (QUANTIFOIL R 1.2/1.3, UT, 200 mesh, EMS cat# Q250-CR1.3-2NM) which had been glow-discharged for 30s at 25 mA using PELCO easiGlow (TedPella Inc.). CryoEM grids were double-blotted and vitrified in liquid ethane using Vitrobot Mark IV (Thermo Fisher) with a blot time of 1s and variable blot forces at 4°C and 100% humidity.

The cryopreserved grids were screened on a FEI Talos Artica microscope at 200 kV equipped with a FEI Falcon3EC detector using the EPU software (Thermo Fisher). The CryoEM data from high quality grids were then collected on a FEI Glacios electron microscope operating at 200 kV, equipped with a Gatan K3 direct electron detector. Micrographs were collected at a magnification of 56,000 corresponding to a pixel size of 0.889 Å with a total exposure dose of 55 or 59 e–/Å^2^.

##### CryoEM data processing, model building and refinement

Motion correction, CTF estimation, particle picking, curation, extraction, 2D classification, ab initio reconstruction, volume refinements and local resolution were performed using CryoSPARC (Structura Biotechnology Inc., Toronto, CA). Particles extracted from a holey carbon grid (Q250-CR1.3 grid) dataset and a holey carbon grid with ultrathin carbon (Q250-CR1.3-2NM) dataset were combined after the particle extraction step in order to provide top and bottom views of the complex. Combined particles were subjected to 2D reference-free classification in CryoSPARC and good 2D classes were selected and subjected to multiple rounds of 2D classification. (Figures S6–S8)

Initial models of BG505 SOSIP from PDB ID 6CDI, PGT121 from PDB ID 5CEZ and N49P9.3-FR from PDB ID 7SX7 (this manuscript) were used as templates. Initial model-to-map fitting cross-correlation was carried out in UCSF ChimeraX.^88^ Several rounds of model refinement were carried out in Phenix (real-space refinement) and Coot (manual refinement). Geometry validation and evaluation were performed by EM-Ringer and Molprobity. Resolution of the structure was further assisted by published crystallographic information positioning the PGT 121 Fab on the BG505 SOSIP.664 trimer^105^ (Table S7). The final model was refined to acceptable geometry with a Chimera CC score of 0.71. The statistics of data collection, reconstruction and refinement are described in Table S6, and the structure has been deposited in the PDB with accession code 7UOJ.

#### *In vivo* testing

##### PK and half-life testing

PK testing was carried out in FcRn−/− hFcRn transgenic mice (Jackson Laboratory). Mice (9 weeks old, equal male and female) were injected intraperitoneally (IP) with 10mg/kg of antibody, and 7 serial blood draws at 0 hours, 8 hours, and days 1, 3, 7, 10,14, 21, and 28 days and IgG Elisa specific for Human IgG measured. Non-compartmental analysis (NCA) was used to calculate the mean of the individual-level terminal half-life estimates for each animal and mAb. Concentrations below the lower limit of the assay were excluded from the analysis. The NCA approach estimates the slope of the terminal elimination phase (λz), where λz was calculated via a linear regression between Y=log(concentrations) and X=time after injection. The time interval was fixed to use all available time points starting with day 3 post-injection (Table S5).

##### Generation of humanized mice

For generation of humanized CD34 mice, newborn mice from strains NSG-SGM3 or NSG-IL15 were exposed to total body radiation (100 cGy) per mouse in a RS-2000 x-ray radiator, followed by intrahepatic injection of 10^5^ CD34^+^ cells under anesthesia (ice for ~ 3 minutes until gross movements cease). Human CD34^+^ cells, isolated from cord blood, were purchased from Lonza (Walkersville, MD). On week 12 after transplantation, mice were checked for human cell reconstitution by double staining with human FITC-conjugated anti-human CD45 antibody and APC-conjugated anti-mouse CD45 antibody (BD Pharmingen). Since reconstitution levels CD45, CD4 and/or NK populations can vary according to donor, animals from each cohort randomized among treatment groups. Only animals exhibiting ≥ 20% total human CD45^+^ cells in circulation were used. Mice successfully transplanted were chosen for further experiments, and designated CD34-NSG-SGM3 or CD34-NSG-IL15 mice.

##### Immunoprophylaxis study

CD34-NSG-SGM3 and CD34-NSG-IL15 mice were injected with various doses of bnAbs or Synagis. Three hours later, mice were bled for measurement of bnAb levels and, immediately after bleeding, challenged with minimum doses of transmitter founder HIV-1 1086c required for 100% infection of control animals. These doses were 30 and 75 TCID_50_ units of a virus stock titrated in primary PBMCs for CD34-NSG-SGM3 and CD34-NSG-IL15 mice, respectively. Plasma HIV RNA was quantified by Taqman qPCR at weekly intervals from Week 1 to week 3. Binary outcome, HIV infection (yes or no) at week 3, is modeled by logistic regression model which includes dose and type of mice as independent variables.

##### Plasma HIV RNA quantification by TagMan qPCR

Plasma viremia was measured using TaqMan Fast Virus 1-Step master mix (Thermo Fisher) in an automated Step One Plus Real-Time PCR detection system (Bio-Rad). The assay uses the previously described HIV primer pair 6F/84R and Taqman probe.^85^ Viral RNA was isolated using Qiagen Viral RNA mini kit. This protocol is intended for isolation of viral RNA from plasma volumes of at least 140 μl. TaqMan qPCR amplification of HIV RNA isolated from 140 μl of patient’s plasma has a limit of detection (LOD) of 40 copies/ml. In humanized mice, available plasma volumes are small, typically ~ 40 μl, requiring a 3.5 dilution with PBS to reach the minimum 140 μl needed for RNA isolation. Accordingly, the LOD of the TaqMan qPCR assay in mouse plasma samples is ~ 150 copies/ml.

### QUANTIFICATION AND STATISTICAL ANALYSIS

The immunoprophylaxis study has 28 CD34-NSG-SGM3 and 29 CD34-NSG-IL15 mice injected with various doses of bnAbs. The outcome, HIV infection is modeled by logistic regression with dose of bnAbs and type of mice as independent variables. By assuming a squared multiple correlation between dose and type of mice=0.0578 and infection rate of CD34-NSG-SGM3 mice=0.54, the study has >80% power to detect 0.098 odds ratio (OR) of CD34-NSG-IL15 to CD34-NSG-SGM3 mice being infected. The logistic regression analysis was performed by using SAS software version 9.4. (SAS Institute Inc., Cary, NC), specifically through PROC LOGISTIC procedure. X-ray crystallography data collection and refinement statistics are summarized in Table 2. Cryo-EM data collection and refinement statistics are shown in Table S6.

## Supplementary Material

MMC1

## Figures and Tables

**Table 1.** Estimated half-life by mAb by non-compartmental analyses

	Half-life (days)
bnAb/mAb (intraperitoneal injection)	hFcRn mice
N49P9.6	4.7
N49P9.6-FR	8.2
N49P9.6-FR-LS	12.0
**Non-Fc-engineered controls**	
VRC01	8.3
3BNC117	7.5
**Fc-engineered controls**	
VRC07-523-LS	6.3
VRC07-523-FR-LS	8.7
VRC01-LS	9.7

**Table 2.** Crystallographic data collection and refinement statistics

	N49P9.1 Fab-gp120_93TH057_ core_e_	N49P9.3 Fab-gp120_93TH057_ core_e_	N49P9.3-FR Fab-gp120_93TH057_ core_e_
**Data collection**			
Wavelength, Å	0.979	0.979	0.979
Space group	I2_1_2_1_2_1_	P1	P1
Cell parameters			
a, b, c, Å	104.8, 110.5, 152.2	62.2, 68.2, 115.1	60.4, 65.4, 112.4
α, β, γ, ◦	90, 90, 90	90.3, 102.3, 90.3	90.0, 104.9, 90.0
Complexes/a.u.	1	2	2
Resolution, (Å)	50–3.15 (3.2–3.15)	50–3.4 (3.46–3.4)	50–2.15 (2.19–2.15)
# of reflections			
Total	59,116	85,037	132,781
Unique	14,779 (746)	25,011 (1,163)	73,767 (2,786)
R_merge_^a,^ %	13.8 (79.4)	11.7 (100)	13.5 (64.5)
R_pim_^b^, %	7.2 (41.4)	7.4 (72.5)	13.5 (64.5)
*CC*_*1*/*2*_^c^	0.99 (0.65)	0.99 (0.82)	0.95 (0.75)
I/σ	12.8 (1.0)	15.5 (1.4)	12.8 (1.0)
Completeness, %	94.4 (96.4)	97.6 (92.6)	82.7 (63.6)
Redundancy	4.0 (4.0)	3.4 (2.9)	1.8 (1.4)
**Refinement statistics**			
Resolution, Å	50.0–3.15	50.0–3.4	50.0–2.15
R^d^ %	24.7	24.0	22.9
R_free_^e^, %	30.0	29.2	26.8
# of atoms			
Protein	5,662	11,710	11,917
Water	–	–	641
Ligand/ion	155	285	592
Overall B value (Å)^2^			
Protein	121	154	45
Water	–	–	46
Ligand/ion	141	157	51
RMSD^f^			
Bond lengths, Å	0.003	0.011	0.004
Bond angles, °	0.72	1.24	0.73
Ramachandran^g^			
Favored, %	89.85	91.9	95.14
Allowed, %	8.9	7.0	4.53
Outliers, %	1.25	1.1	0.33
PDB ID	6OZ3	7SX6	7SX7

Values in parentheses are for highest-resolution shell.

a *R*_merge_ = ∑|*I* - <*I*>|/∑*I*, where *I* is the observed intensity and <*I*> is the average intensity obtained from multiple observations of symmetry-related reflections after rejections.

b R_pim_ = as defined in the study by Weiss.^77^

c *CC*_*1/2*_ = as defined by Karplus and Diederichs.^78^

d *R* = ∑‖F_o_|- | F_c_‖/∑|F_o_ |, where F_o_ and F_c_ are the observed and calculated structure factors, respectively.

e R_free_ = as defined by Brünger.^79^

f RMSD = Root-mean-square deviation.

g Calculated with MolProbity.

**Table:** KEY RESOURCES TABLE

REAGENT or RESOURCE	SOURCE	IDENTIFIER
**Antibodies**		
N49P9	This study	N/A
N49P9.1	This study	N/A
N49P9.3	This study	N/A
N49P9.6	This study	N/A
N49P9.3-FR	This study	N/A
N49P9.6-FR-LS	This study	N/A
eN49P9	This study	N/A
PGT121	This study	RRID: AB_2491041
Synagis	Medimmune LLC	RRID: AB_2459638
**Bacterial and virus strains**		
119 HIV-1 pseudovirus “global panel”	(Seaman et al.)^81^; this study	N/A
**Biological samples**		
Human CD34 Cells	Lonza	N/A
**Chemicals, peptides, and recombinant proteins**		
HIV BaL gp120	(Fouts et al.)^82^; this study	N/A
HIV BG505 SOSIP.664	(Sanders et al.)^83^; this study	N/A
HIV A/E gp120_93TH057_core_e_	(Acharya et al.2014)^84^; this study	N/A
HIV CH505TFchim.6R.SOSIP.664v4.1	Dr. Barton Haynes, Duke University	N/A
**Critical commercial assays**		
Protein A agarose	GE Healthcare	Cat#20333
TaqMan Fast Virus 1-Step master mix	Thermo Fisher	Cat#4444432
**Deposited data**		
Crystal Structure N49P9.1	This study	PDB: 6OZ3
Crystal Structure N49P9.3	This study	PDB: 7SX6
Crystal Structure N49P9.3-FR	This study	PDB: 7SX7
Cryo-EM Structure PGT121 and N49P9.6-FR3	This study	PDB: 7UOJ
Cryo-EM map of PGT121 and N49P9.6-FR3	This study	EMDB: EMD-26648
Sequence of N49P9	^26^, This study	GenBank:MG819641 (heavy chain) MG819646 (light chain)
Sequence of N49P9.3	This study	GenBank:PV344714.1 (light chain) PV344713.1 (heavy chain)
Sequence of PGT121	^80^, This study	GenBank:JN201911 (light chain) JN201894 (heavy chain)
**Experimental models: Cell lines**		
TZM-bl cells	NIH AIDS Reagent Program	Cat#8129
Freestyle 293-F cells	Thermo Scientific	Cat #R79007
CEM.NKR_CCR5_ cells	Dr. John Kappes	N/A
HD-BIOP3 GS Null CHO K1	Revvity	N/A
**Experimental models: Organisms/strains**		
*scid* FcRn−/− hFcRn	Jackson Laboratories	Strain #018441
NSG-SGM3	Jackson Laboratories	Strain #013062
NSG-IL15	Jackson Laboratories	Strain #030890
**Oligonucleotides**		
6F 5’- CAT GTT TTC AGC ATT ATC AGA AGG A -3’	Palmer et al.^85^	N/A
84R 5’-TGC TTG ATG TCC CCC CAC T-3’	Palmer et al.^85^	N/A
FAM-P 5’- /56-FAM/CCA CCC CAC /ZEN/AAG ATT TAA ACA CCA TGC TAA /31ABkFQ/-3’	Palmer et al.^85^	N/A
**Software and algorithms**		
Prism (v5.0)	GraphPad	http://www.graphpad.com
CryoSPARC	Structural Biotechnology Inc.	https://cryosparc.com
REFMAC	Murshudov et al.^86^	http://www.ccp4.ac.uk/html/refmac5/description
PHENIX	Adams et al.^87^	https://www.phenix-online.orq
ChimeraX	Meng et al.^88^	https://www.rbvi.ucsf.edu/chimerax
COOT	Guan et al.^89^, University of Cambridge	https://www2.mrc-lmb.cam.ac.uk/personal/pemsley/coot/
MolProbity	Davis et al.^90^, Duke University	molprobity.biochem.duke.edu
*PPP4 Suite*	Collaborative Computational Project^91^, Science and Technology Facilities Council	www.ccp4.ac.uk/
PyMol Molecular Graphic suite	DeLano Scientific	http://pymol.org
PISA Webserver	Protein Data Bank in Europe^77^	www.ebi.ac.uk/pdbe/pisa
SAS software version 9.4	SAS Institute Inc.	https://www.sas.com

## Highlights

- Engineered bnAb N49P9.6-FR-LS shows best-in-class breadth, potency, and half-life
- N49P9.6-FR-LS also combines well with bNAbs of other classes
- Final variant eN49P9 is optimized for clinical-grade cGMP manufacturing
- Enhanced bnAbs demonstrate strong antiviral potential and translational promise
