# Supplementary material for: A comprehensive engineering strategy improves potency and manufacturability of a near pan-neutralizing antibody against HIV
Source: Structure. Author manuscript; Available in PMC 2025 Jul 5. (PMC12227296; doi:10.1016/j.str.2025.04.016)
Supplement: MMC1 [file NIHMS2077715-supplement-MMC1.pdf]

Structure, Volume *33*

Supplemental Information

A comprehensive engineering strategy improves potency and manufacturability of a near pan-neutralizing antibody against HIV

Mohammad M. Sajadi, Abdolrahim Abbasi, Zahra Rikhtegaran Tehrani, Christine Siska, Rutilio Clark, Woo Chi, Michael S. Seaman, Dieter Mielke, Kshitij Wagh, Qingbo Liu, Taylor Jumpa, Randal R. Ketchem, Dung N. Nguyen, William D. Tolbert, Brian G. Pierce, Ben Atkinson, Derrick Deming, Megan Sprague, Andrew Asakawa, David Ferrer, Yasmin Dunn, Sarah Calvillo, Rui Yin, Johnathan D. Guest, Bette Korber, Bryan T. Mayer, Alicia H. Sato, Xin Ouyang, Scott Foulke, Parham Habibzadeh, Maryam Karimi, Arash Aslanabadi, Mahsa Hojabri, Saman Saadat, Roza Zareidoodeji, Mateusz Kędzior, Edwin Pozharski, Alonso Heredia, Hegang Chen, David Montefiori, Guido Ferrari, Marzena Pazgier, George K. Lewis, Joseph G. Jardine, Paolo Lusso, and Anthony DeVico

A comprehensive engineering strategy improves potency and manufacturability of a near pan-neutralizing antibody against HIV

Mohammad M. Sajadi,[1,2,*] Abdolrahim Abbasi,[1] Zahra Rikhtegaran Tehrani,[1] Christine Siska,[3] Rutilio Clark,[3] Woo Chi,[3] Michael S. Seaman,[4] Dieter Mielke,[5] Kshitij Wagh,[5] Qingbo Liu,[6] Taylor Jumpa,[3] Randal R. Ketchem,[3] Dung N. Nguyen,[7] William D. Tolbert,[7] Brian G. Pierce,[8,9] Ben Atkinson,[1] Derrick Deming,[3] Megan Sprague,[3] Andrew Asakawa,[3] David Ferrer,[3] Yasmin Dunn,[3] Sarah Calvillo,[3] Rui Yin,[8,9] Johnathan D. Guest,[8,9] Bette Korber,[10] Bryan T. Mayer,[11] Alicia H. Sato,[11] Xin Ouyang,[1] Scott Foulke,[1] Parham Habibzadeh,[1] Maryam Karimi, [1] Arash Aslanabadi,[1] Mahsa Hojabri,[1] Saman Saadat,[1] Roza Zareidoodeji,[1] Mateusz Kędzior,[12] Edwin Pozharski,[8,13] Alonso Heredia,[1] Hegang Chen,[1] David Montefiori,[14] Guido Ferrari,[5,14] Marzena Pazgier,[7] George K. Lewis,[1] Joseph G. Jardine,[15] Paolo Lusso,[16] Anthony DeVico[1]

1 Divisions of Vaccine Research and Clinical Care and Research, Institute of Human Virology, University of Maryland School of Medicine, Baltimore, MD, USA

2 Department of Medicine, Maryland VA Healthcare System, Baltimore, MD, USA

3. Just-Evotec Biologics, 401 Terry Ave. North, Seattle, WA, USA

4. Center for Virology and Vaccine Research, Beth Israel Deaconess Medical Center, Harvard Medical School, Boston, MA 02115, USA

5. Duke Human Vaccine Institute, Durham, NC, USA

6. Key Laboratory of Developmental Genes and Human Disease, School of Life Science and Technology, Southeast University, Nanjing, China

7. Infectious Disease Division, Department of Medicine, Uniformed Services University of the Health Sciences, Bethesda, MD, USA

8. University of Maryland Institute for Bioscience and Biotechnology Research (IBBR), Rockville, MD, USA

9. Department of Cell Biology and Molecular Genetics, University of Maryland, College Park, MD, USA

10. Theoretical Biology & Biophysics, Los Alamos National Laboratory, Los Alamos, NM, USA

11. Vaccine and Infectious Disease Division, Fred Hutchinson Cancer Center, Seattle, WA, USA

12. Neutralizing Antibody Center, IAVI, La Jolla, CA, USA

13. Department of Biochemistry and Molecular Biology, University of Maryland School of Medicine, Baltimore, MD, USA 14. Division of Surgical Sciences, Duke University School of Medicine, Durham, NC, USA

15. Department of Immunology and Microbiology, Scripps Research Institute, La Jolla, CA, USA

16. Laboratory of Immunoregulation, National Institute of Allergy and Infectious Diseases, NIH, Bethesda, Maryland, USA

* Corresponding author and lead contact

Email: msajadi@ihv.umaryland.edu

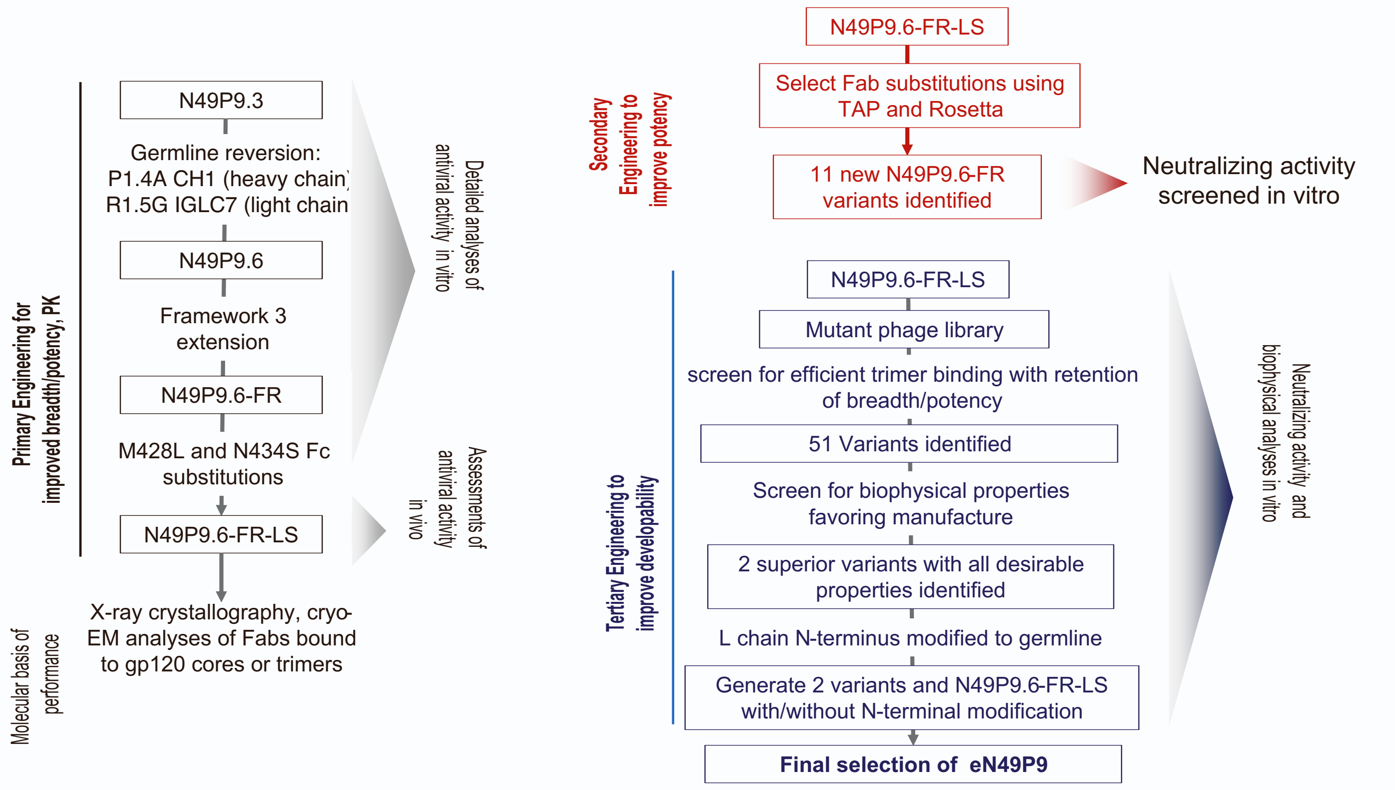

Figure S1. Flow chart of eN49P9 engineering strategy, Related to STAR Methods.

Virus ID	N49 plasma ID50	N49 plasma ID80
CNE58	1,078	467
HIV-001428-2.42	1,430	546
CNE52	984	379
6480.v4.c25	1,317	281
Q842.d12	591	170
6041.v3.c23	316	88
Q259.d2.17	521	193
ZM109F.PB4	328	91
235-47	1,519	355
Q23.17	1,810	764
Du156.12	1,025	352
R2184.c04	496	184
0815.v3.c3	173	<40
3365.v2.c2	2,504	535
3415.v1.c1	677	186
BJOX028000.10.3	2,249	346
HIV-16055-2.3	830	346
Ce0682_E4	774	200
RHPA4259.7	496	79
C2101.c01	1,378	246
ZM249M.PL1	1,228	255
TRO.11	578	102
WITO4160.33	993	185
Ce0393_C3	345	116
249M B10	296	95
SC422661.8	1,095	289
R1166.c01	552	184
C3347.c11	1,943	252
X1193_c1	1,772	405
191084 B7-19	3,724	175
Q769.d22	258	94
6952.v1.c20	1,006	268
BJOX025000.01.1	427	125
X1254_c3	568	208
3016.v5.c45	709	271
BF1266.431a	498	169
WEAU_d15_410_787	1,091	321
CNE19	512	113
3301.v1.c24	457	151
P0402_c2_11	783	202
9004SS_A3_4	97	<40
MS208.A1	164	48
263-8	641	170
231966.c02	783	133
BJOX015000.11.5	422	59
3103.v3.c10	172	63
CNE53	234	52
X1632_S2_B10	324	87
TRJO4551.58	687	100
X2088_c9	398	67
CAP45.2.00.G3	174	43
C4118.c09	403	85
REJO4541.67	877	150
ZM247v1(Rev-)	925	244
T255-34	688	244
Du172.17	133	46
CNE8	78	<40
211-9	356	134
6811.v7.c18	354	83
1012_11_TC21_3257	295	41
Q461.e2	NT	NT
Ce2010_F5	233	40
PVO.4	1,005	295
6244_13_B5_4576	600	97
T251-18	233	54
QH0692.42	274	60
CNE21	199	68
Ce703010054_2A2		
Ce1086_B2	534	139
ZM214M.PL15	491	170
X2131_C1_B5	543	143
1006_11_C3_1601	879	293
928-28	386	83
0260.v5.c36	178	50
A07412M1.vrc12	401	93
R3265.c06	251	63
CNE20	159	57
CNE5	263	83
ZM197M.PB7	146	<40
CNE30	355	90
Ce704809221_1B3	314	51
1056_10_TA11_1826	318	78
Ce2060_G9	250	56
SC05_8C11_2344	207	71
89-F1_2_25	680	148
ZM233M.PB6	837	155
C1080.c03	852	106
62357_14_D3_4589	172	<40
AC10.0.29	363	112
HIV-0013095-2.11	230	54
6240_08_TA5_4622	160	<40
T257-31	139	43
Ce1176_A3	114	<40
CNE17	327	52
BJOX010000.06.2	330	105
7030102001E5(Rev-)	330	96
P1981_C5_3	54	<40
6405.v4.c34	293	91
231965.c01	127	<40
BJOX009000.02.4	515	161
ZM53M.PB12	136	46
1054_07_TC4_1499	727	240
1394C9G1(Rev-)	163	<40
246F C1G	230	59
Du422.1	121	42
HIV-16845-2.22	254	52
ZM135M.PL10a	438	68
6535.3	460	93
3817.v2.c59	1,140	246
Ce1172_H1	112	<40
CAAN5342.A2	342	103
THRO4156.18	144	46
191955_A11	151	44
CAP210.2.00.E8	104	<40
T250-4	631	167
T278-50	45	<40
620345.c01	<40	<40
6540.v4.c1	766	245
6545.v4.c1	165	49

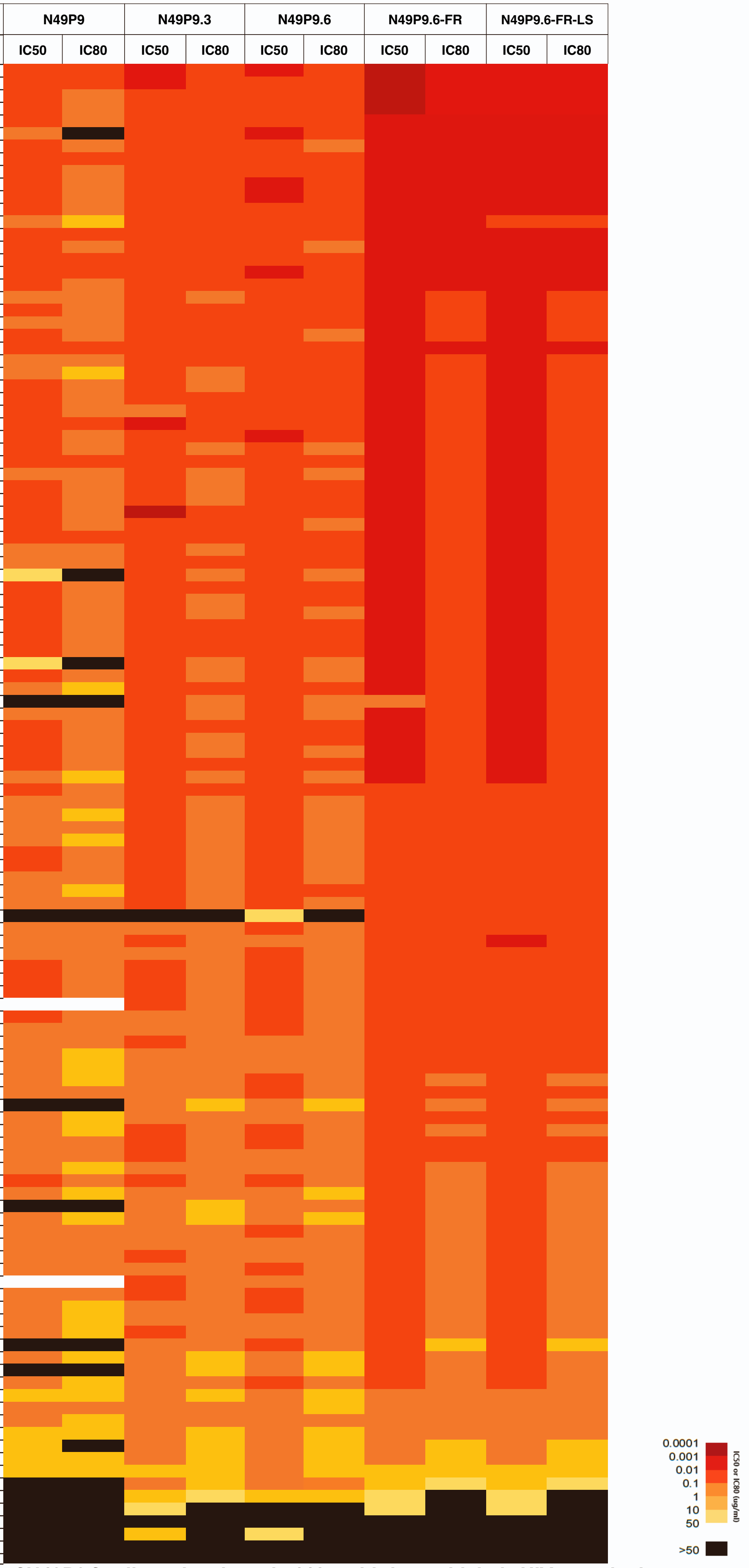

Figure S2. **Neutralization testing of N49P9 family and variants in 118 multi-tier multiclade HIV pseudovirus assay, Related to Figure 2, and STAR Methods.** IC50 and IC80 values given as colors and heat map (refer to color key).

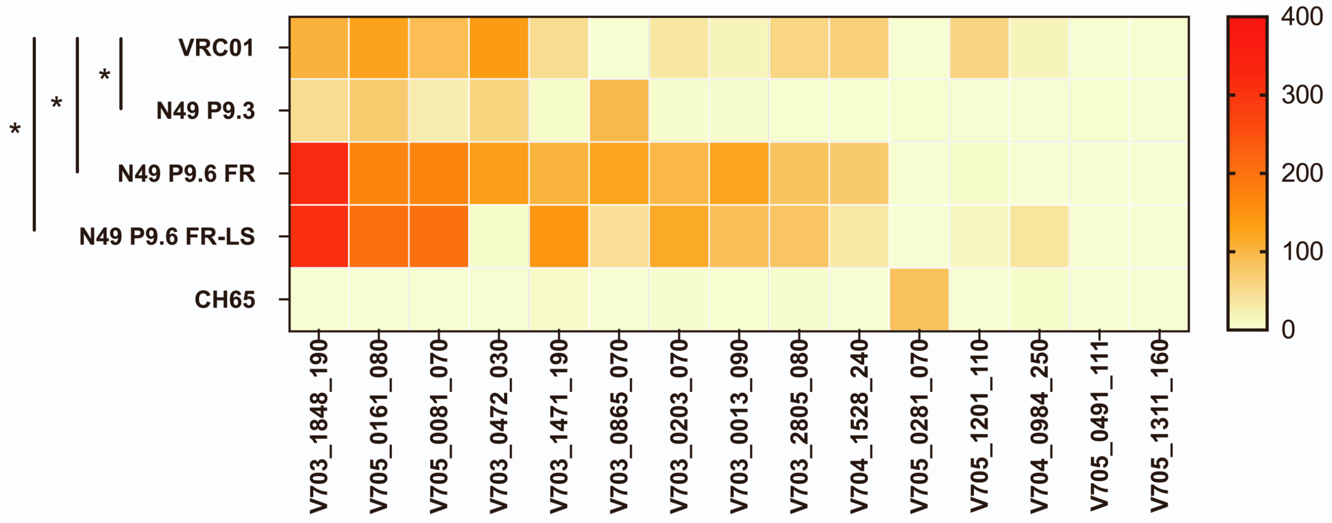

Figure S3, ADCC activity of N49 P series variants, Related to STAR Methods. The heat map represents the magnitude of responses against infectious molecular clones expressing recent Envelopes from the placebo group of phase II/III trials (HVTN703/704/705) as area under the curve (AUC) from 0 (no activity) to ≥400 (highest) as indicated by the scale on the right y-axis. Antibody-specific killing of each mAb listed on the left y-axis was conducted against each HIV IMC (x-axis) starting at 50µg/mL using a 5-fold dilution. AUCs were then calculated using the trapezoid rule after subtracting the background activity. The anti-flu CH65 mAb was used as negative control. The parental antibody mAb N49P9.3 displayed significantly less ADCC activity than VRC01, while the engineered variants N49P9.6-FR, and N49P9.6-FR-LS demonstrated significantly more ADCC activity than VRC01, when tested by paired t test. * = $P < .05$

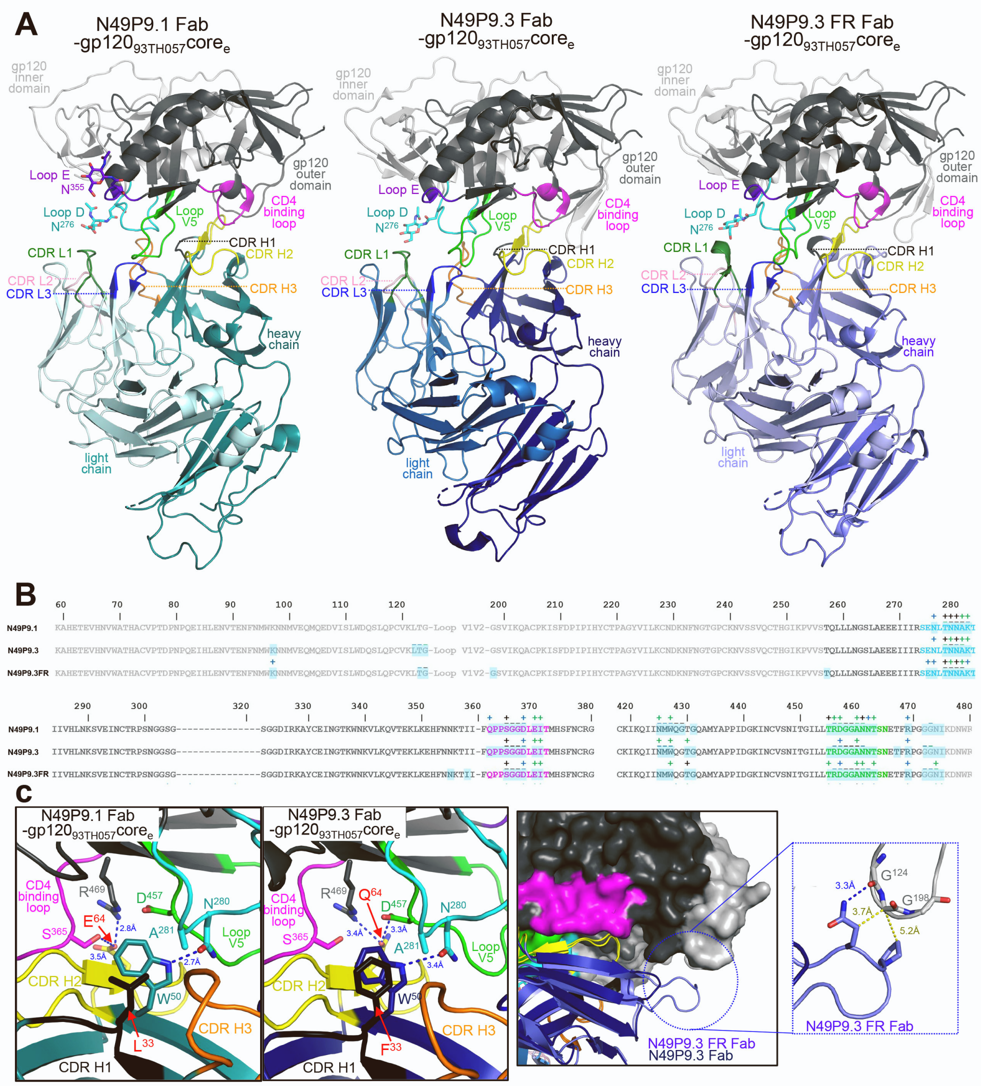

Figure S4. Crystal structures of N49P9.1Fab-, N49P9.3-Fab- and N49P9.3FR Fab-gp120$_{93TH057}$core$_e$ complexes, Related to Table 2 and STAR Methods. (**A**) The overall structure of the complexes are shown as ribbon diagrams. The complementary-determining regions (CDRs) of Fabs are colored: CDR L1, green; CDR L2 light pink; CDR L3, blue; CDR H1, black; CDR H2, yellow and CDR H3, light orange. Outer and inner domains of gp120 are dark and light grey, respectively. The outer domain loops: D, E, CD4 binding, and V5 are colored in cyan, purple blue, magenta, and light green, respectively. Carbohydrates at position N^{276} (loop D) and N^{355} (loop E) are shown as sticks. (**B**) Epitope footprints of Fabs N49P9.1 and N49P9.3 mapped onto the gp120 primary sequences. Contact residues are defined by a 5 Å cutoff and marked above the sequence with (+) for side chain and (-) for main chain to indicate the type of contact: hydrophilic (blue), hydrophobic (green) and both (black). Buried surface residues determined by PISA are shaded blue for primary (**C**) Details of N49P9.3 Fab-gp120$_{93TH057}$core$_e$ interface with a blow-up view into the Fab contacts mediated by CDR H1 and 2 of N49P9.1 and N49P9.3 with colors indicated as in (A) and hydrogen bonds shown as dotted blue lines *(left panel)*. Interaction network of the frame region of N49P9.3-FR to gp120$_{93TH057}$core$_e$ *(right panel)*. H-bonds and Van der Waals contacts are shown as blue and yellow dotted lines, respectively. Residues that differ between N49P9.1 and N49P9.3 are labeled in red.

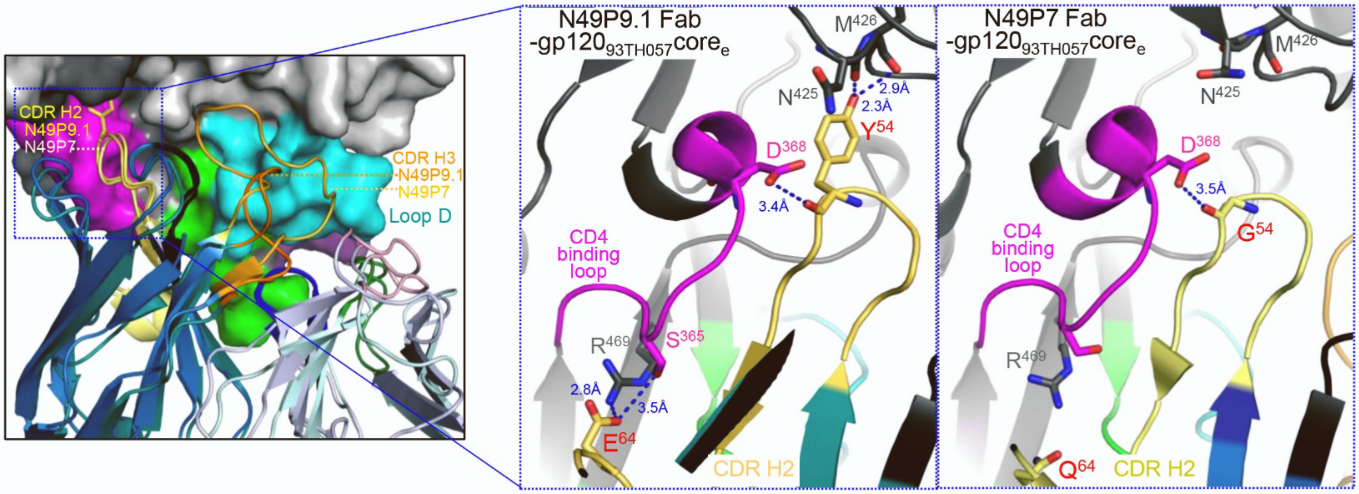

Figure S5. Comparisons of Fab-gp120$_{93TH057}$core$_e$ interfaces between N49P9.1 and N49P7, **Related to Table 2 and STAR Methods.** Structures of N49P9.1 Fab-gp120$_{93TH057}$core$_e$ and N49P7 Fab-gp120 $_{93TH057}$core$_e$ (PDB code: 6BCK) were superimposed based on the gp120 core. Fab CDRs and gp120 are colored as in Figure S3. The blow-up view shows specific contacts mediated by the CDR H2 of both Fabs. H-bonds are shown as blue dotted lines and residues that differ between N49P9.1 and N49P7 are labeled in red.

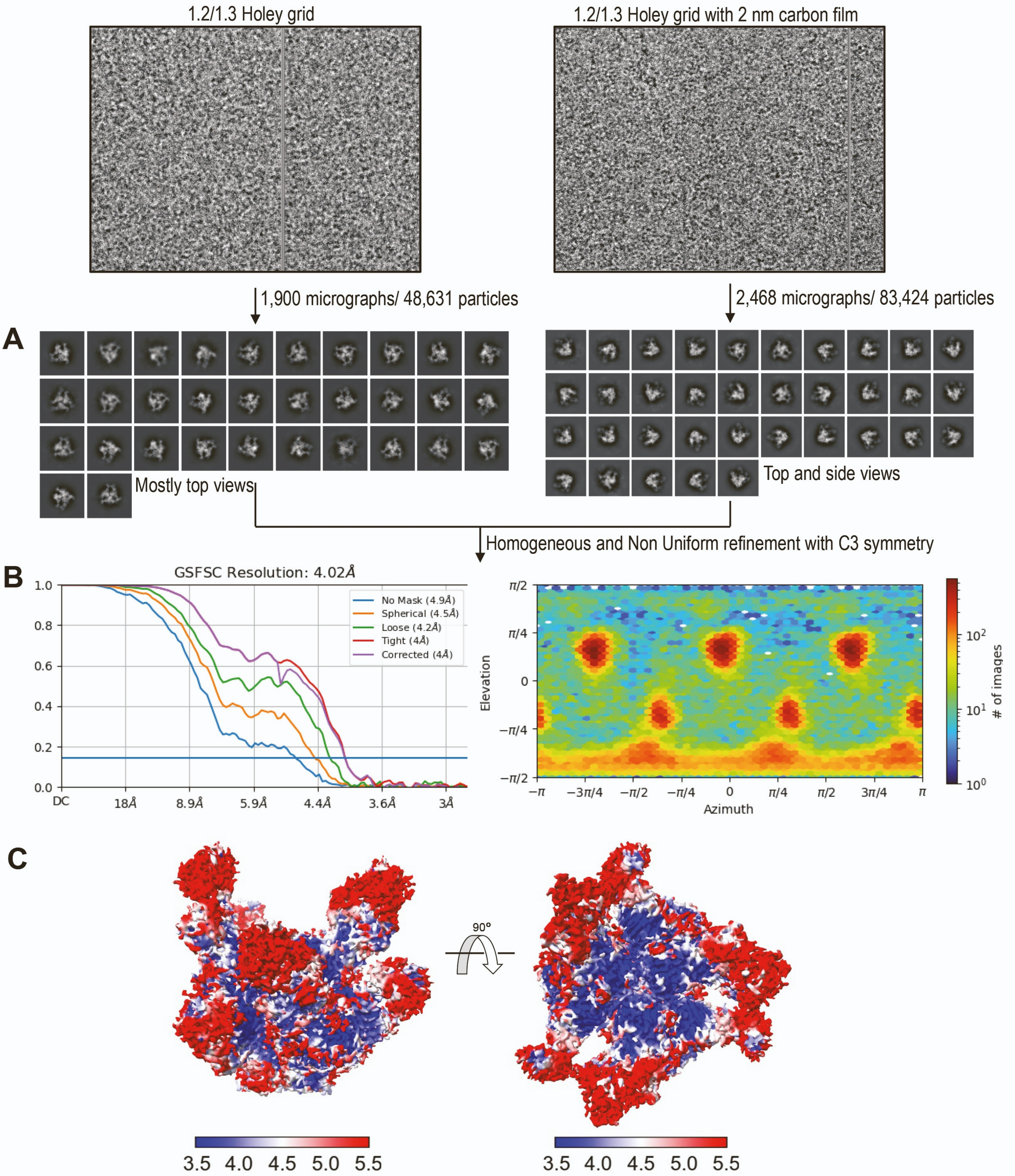

Figure S6. Cryo-EM structure of BG505 SOSIP.664 HIV-1 bound complex N49P9.6-FR and PGT121, Related to Figure 5 and STAR Methods. (**A**) Micrographs from two different types CryoEM grids were used for the reconstruction. A standard Quantifoil 1.2/1.3 grid gave mainly top views of the complex while Quantifoil 1.2/1.3 grids with a 2 nm carbon layer gave both top and side views. Particles from selected 2D classes from both grids were then used for *ab initio* map reconstruction. (**B**) The Fourier shell correlation curves with spherical mask indicate the overall resolution (FSC cutoff 0.143) as determined by CryoSPARC and the direction distribution plot of all particles used in the final refinement. (**C**) Local resolution estimation plots with top and side views of the complex colored from blue, higher resolution, to red, lower resolution as indicated.

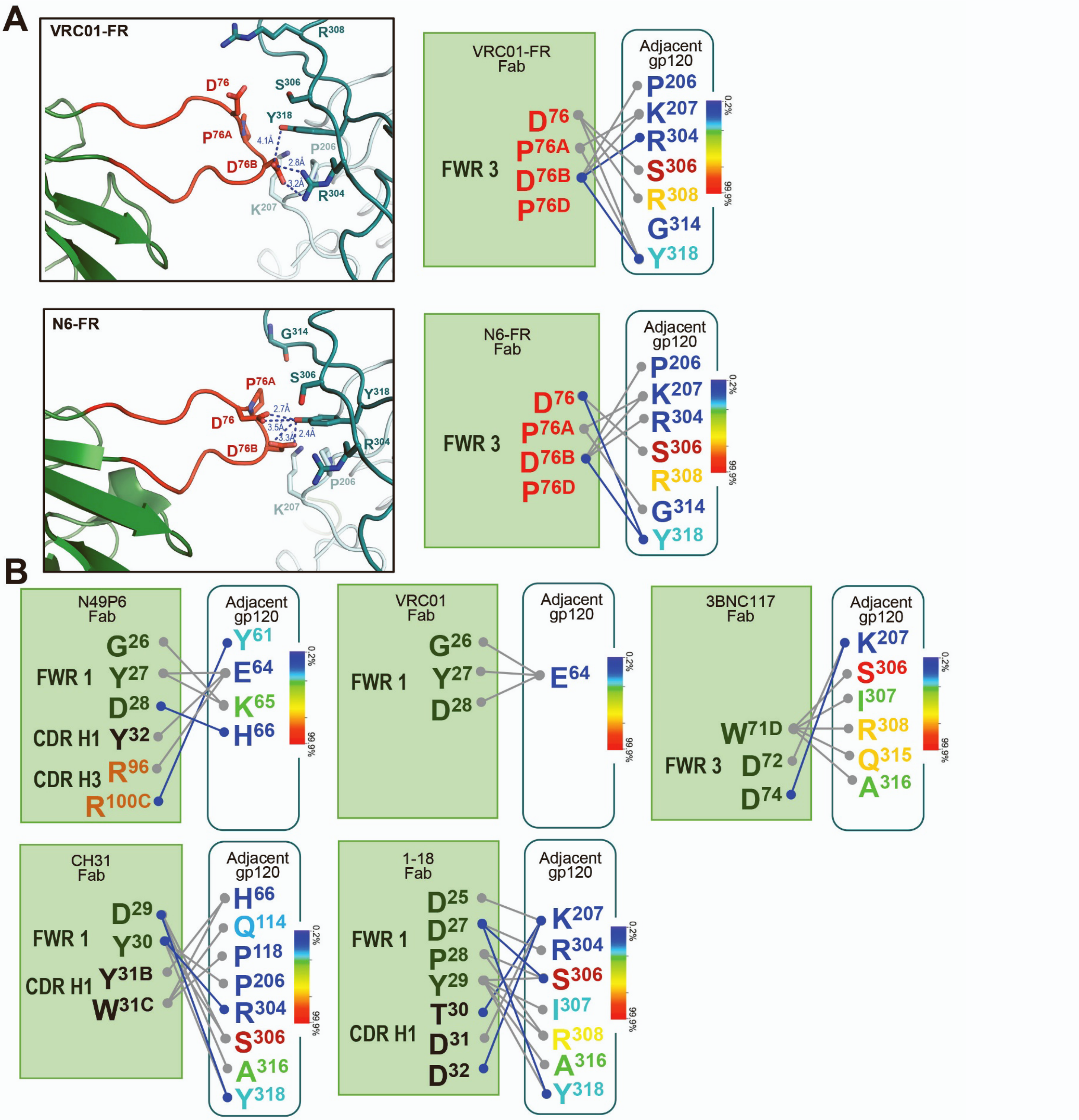

Figure S7. Interaction network of CD4-binding site bnAbs with the adjacent gp120 protomer, Related to Figure 5, and STAR Methods. (**A**) (*left*) Blow up views into the VRC01-FR and N6-FR interaction network to the adjacent gp120 protomer shown from PDB codes 6NNF and 6NM6, respectively. Hydrogen bonds (<4.1Å) are shown as blue dashes. (*right*) The Fab-adjacent gp120 contacts shown to highlight interacting residues. Hydrophobic contacts less than 5 Å are shown with gray lines and hydrogen bonds less than 4.1 Å are shown with blue lines. The gp120 residues are color-coded in a gradient based upon sequence conservation: dark blue corresponds to the percentage of sequences in the HIV sequence compendium (https://www.hiv.lanl.gov/content/sequence/HIV/COMPENDIUM/compendium.html) that differ at that position from the Hxbc2 reference sequence 0.2-7% of the time and red corresponds those that differ at that position from Hxbc2 87-99.9% of the time. Intermediate colors correspond to intermediate percentages on a roughly linear scale. Only protein-protein contacts are shown. (**B**) The Fab-adjacent gp120 contacts of bnAbs N49P6, VRC01, 3BNC117, CH31 and 1-18 (PDB codes: 6OZ4, 5FYJ, 5V8M, 6NNJ, and 6UDJ) shown with line colors as in (**A**).

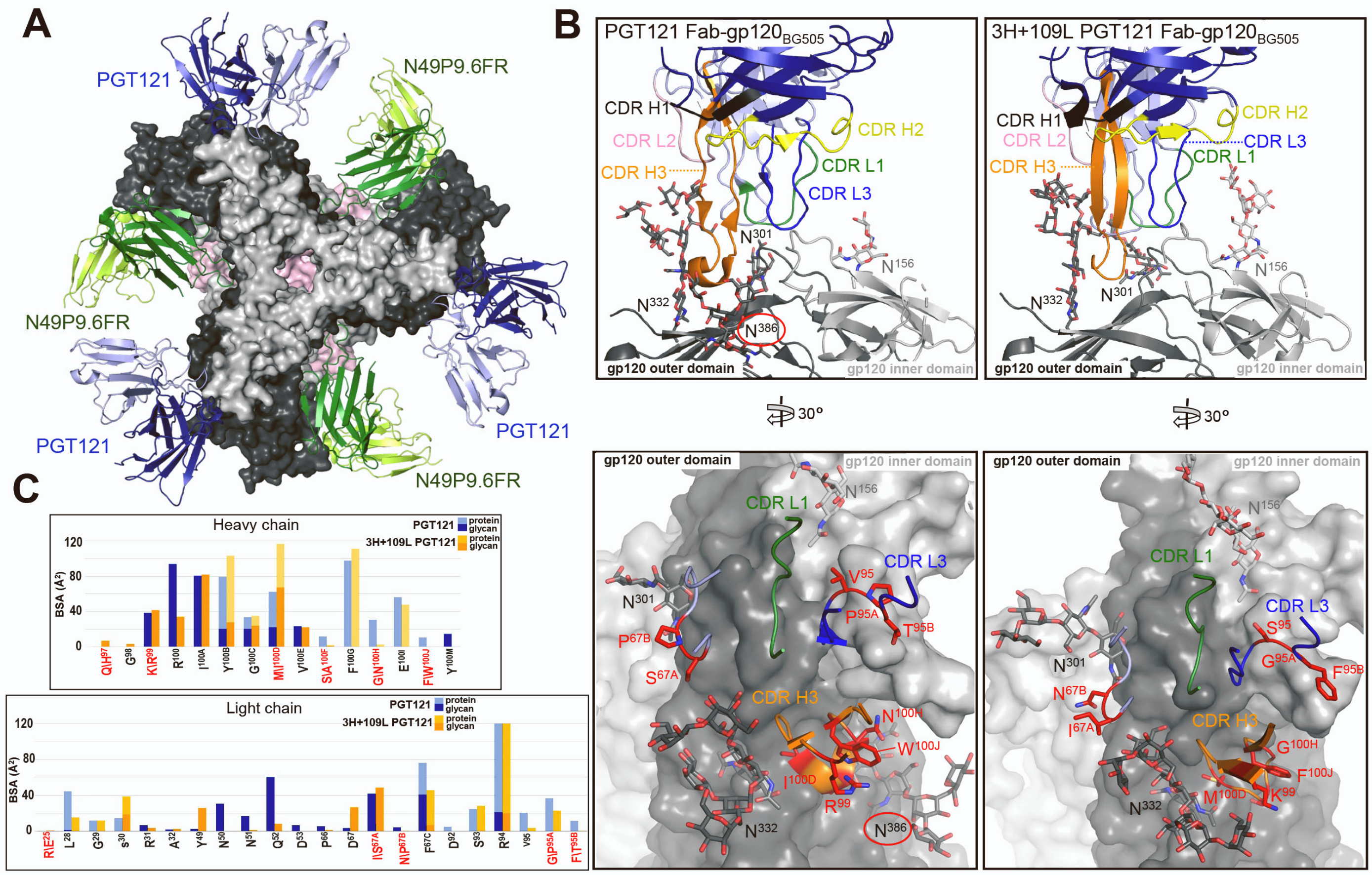

Figure S8. Molecular details of PGT121 bound to BG505 SOSIP.664, Related to Figure 5 and STAR Methods. (**a**) Top view of the complex of BG505 SOSIP.664 HIV-1 Env trimer with N49P9.6-FR and PGT121 bound. N49P9.6-FR Fabs and gp120 are colored as in Figure 3. PGT121 (only the variable regions of the Fabs were built into the Cryo-EM model) are shown in blue and light blue ribbons. (**b**) Side-by-side comparison of PGT121 and the inferred germline PGT121 precursor, 3H+109L PGT121 (PDB ID: 5CEZ). CDRs are colored as in Figure 2. (**c**) BSA plot of Fab residues contributing to binding for PGT121 and 3H+109L PGT121. Protein or glycan portions are as indicated with residues that differ in sequence in red, 3H+109L PGT121 residue bottom and PGT121 residue top. One potential additional glycan interaction between PGT121's light chain and the glycan attached to N^{386} that is absent in 3H+109L PGT121 is encircled in red in PGT121. The gp120 contact residues are largely identical between PGT121 and 3H+109L PGT121 but interactions PGT121 are more protein dependent. The total BSA for 3H+109L PGT121 is 2275 $Å^2$ (1211 $Å^2$ for gp120 and 1064 $Å^2$ for Fab); total BSA for PGT121 is 2455 $Å^2$ (1236 $Å^2$ for gp120 and 1219 $Å^2$ for Fab). Interactions between protein residues explain the total BSA of 1126 $Å^2$ for 3H+109L PGT121 and the total BSA of 1308 $Å^2$ for PGT121. Contributions from glycan are roughly comparible for both, 1126 $Å^2$ for 3H+109L PGT121 and 1147 $Å^2$ for PGT121. Thus one way PGT121 seems to have increased its affinity to Env during maturation is to have maximized its interactions with protein residues while maintaining similar interactions with key glycans.

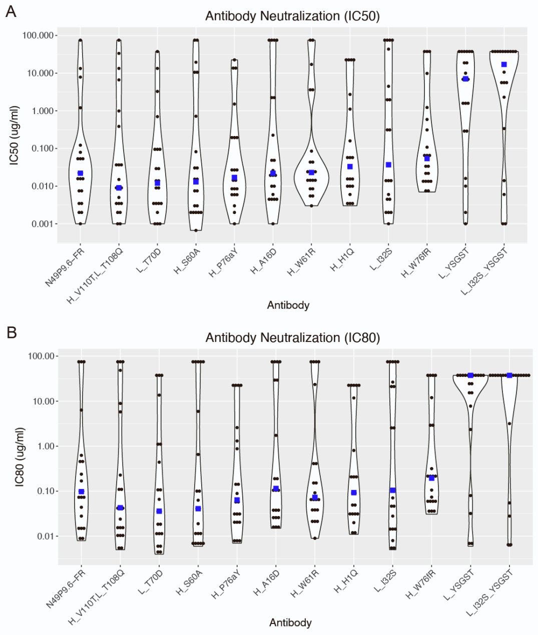

Figure S9. Neutralization assessment of N49P9.6-FR designs, Related to STAR Methods. Designed antibodies and N49P9.6-FR wild-type were tested for neutralization using a 21 virus global HIV panel, and measured (A) IC50 and (B) IC80 levels are shown as violin plots. Individual neutralization values are shown as black points, with median values shown as blue squares, and designs are ordered from lower to higher median IC50 levels (with wild-type on left for reference). In cases with unquantified neutralization, neutralization values are represented as 1.5-fold versus the unquantified level (e.g. 75 µg/ml for >50 µg/ml). Figure generated with ggplot2[1].

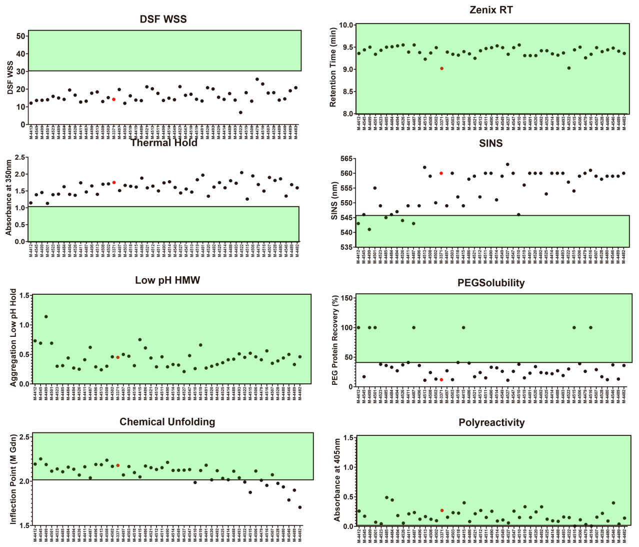

Figure S10. Conformational and colloidal stability results for variants selected from library panning, Related to STAR Methods. Regions of generally acceptable developability results are highlighted in green, N49P9.6-FR-LS are indicated in red circles compared with variants in black circles. A change in conformational stability was not observed while an increase colloidal stability was observed for multiple variants. Each dot represents one individual variant.

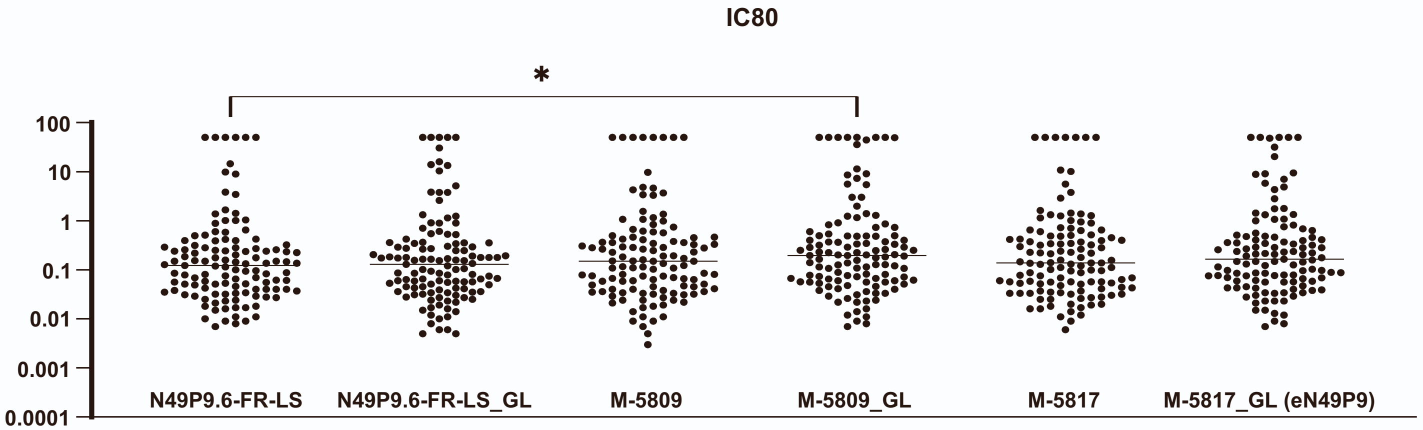

Figure S11. Neutralizing activity of N49P9.6-FR-LS variants selected for manufacturability characteristics, Related to STAR methods. Six selected variants (see narrative) were expressed from stable cells pools and tested for neutralization against a 119 multi-tier multi-clade neutralization panel. All the variants tested had less than two fold difference in median IC80 compared to the parental N49P9.6-FR-LS, although this was significant only for M-5809_GL compared to N49P9.6-FR-LS (P=.04 by Mann-Whitney test). Variant M-5817_GL was the final variant chosen for clinical development (eN49P9). "-GL": use of light chain with uncleaved N-terminus. *= P<.05 by the Mann-Whitney test.

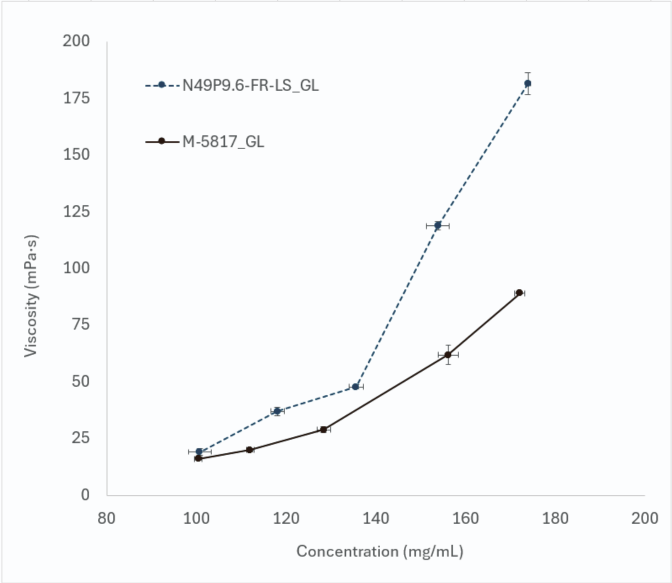

Figure S12. Viscosity measurement of final variant and parental form, Related to STAR Methods. Increased colloidal stability as measured by biophysical analysis was confirmed by viscosity measurement across increasing protein concentration. Decrease in viscosity was observed in the lead candidate, M-5817_GL (black line; also known as eN49P9) as compared with the parental molecule N49P9.6-FR-LS (germline N-term) (blue dotted line). Standard deviation of triplicate protein concentration and viscosity measurements are capture in hash marks for each axis.

Table S1. Single bnAb neutralization characteristics and ranking, Related to Figure 3, and STAR Methods.

bnAb	IC80 geometric mean (µg/ml)	IC80 < 1µg/ml breadth (%)	IC99 geometric mean (µg/ml)	IC99 < 10µg/ml breadth (%)	Log10 IIP at 30µg/ml	IIP > 5Log10 breadth (%)	Average rank	bnAb epitope class
N49P9.6-FR	0.069	88.542	1.057	82.292	3.853	14.583	1.000	CD4bs
VRC01.23LS	0.198	84.375	2.578	78.125	3.274	11.458	1.857	CD4bs
N6	0.326	83.333	3.613	77.083	3.267	5.208	3.286	CD4bs
N49P9.6	0.219	83.333	3.496	73.958	3.045	1.042	3.571	CD4bs
1-18	0.264	80.208	5.506	63.542	2.744	2.083	4.286	CD4bs
VRC07-523-	0.333	79.167	9.072	55.208	2.484	0.000	6.286	CD4bs
PGDM1400	0.348	61.458	5.931	42.708	2.089	1.042	6.286	V2 apex
VRC26.25	0.663	52.083	7.287	35.417	1.273	6.250	6.857	V2 apex
3BNC117	0.941	60.417	12.287	40.625	2.231	1.042	7.143	CD4bs
PGT121	1.642	40.625	10.305	37.500	0.935	1.042	8.429	V3 glycan
10-1074	2.931	42.708	14.892	31.250	0.987	2.083	8.857	V3 glycan
VRC01	1.775	38.542	15.600	34.375	2.096	0.000	9.429	CD4bs

*CD4bs = CD4 binding site

Table S2. Dual bnAb neutralization characteristics and ranking, Related to Figure 3, and STAR Methods.

bnAb	IC80 geometric mean (μg/ml)	IC80 < 1μg/ml breadth	IC80 breadth with 2 bnAbs active	IC99 geometric mean (μg/ml)	IC99 < 10μg/ml breadth (%)	IIP at 30μg/ml (Log10)	IIP > 5Log10 breadth (%)	Average rank
N49P9.6-FR+PGDM1400	0.035	93.750	64.58	0.433	89.583	5.382	55.208	1.857
N49P9.6-FR+VRC26.25	0.029	93.750	47.91	0.407	90.625	4.736	47.917	3.286
VRC01.23LS+PGDM140	0.071	89.583	64.58	0.817	87.500	4.873	47.917	4.143
N49P9.6-FR+PGT121	0.056	91.667	47.91	0.605	90.625	4.622	44.792	4.714
N49P9.6+PGDM1400	0.074	90.625	65.62	0.969	84.375	4.493	41.667	5.714
VRC01.23LS+VRC26.25	0.067	88.542	50.00	0.913	84.375	4.519	40.625	6.714
N6+PGDM1400	0.106	86.458	66.66	1.187	83.333	4.686	44.792	7.714
N49P9.6-FR+10-1074	0.075	91.667	45.83	0.787	89.583	4.295	37.500	8.000
1-18+PGDM1400	0.086	89.583	64.58	1.173	77.083	4.242	40.625	8.857
N49P9.6+VRC26.25	0.068	88.542	48.95	1.080	83.333	3.839	36.458	10.143
N6+VRC26.25	0.100	85.417	51.04	1.282	81.250	3.990	37.500	11.857
VRC01.23LS+PGT121	0.122	87.500	46.87	1.308	83.333	4.135	38.542	11.857
VRC07-523-	0.089	85.417	62.50	1.469	73.958	3.860	35.417	13.714
1-18+VRC26.25	0.082	87.500	50.00	1.369	72.917	3.817	30.208	14.286
N49P9.6+PGT121	0.138	86.458	47.91	1.635	78.125	3.900	31.250	15.429
N6+PGT121	0.201	82.292	48.95	1.912	80.208	4.067	36.458	16.286
VRC01.23LS+10-1074	0.182	85.417	44.79	1.746	82.292	3.897	32.292	17.286
3BNC117+PGDM1400	0.128	79.167	53.12	1.892	73.958	3.879	28.125	18.000
VRC07-523-	0.085	85.417	46.87	1.906	66.667	3.182	30.208	18.571
N49P9.6+10-1074	0.198	87.500	45.83	2.099	80.208	3.695	28.125	18.857
N6+10-1074	0.304	81.250	46.87	2.469	78.125	4.023	35.417	19.857
VRC01+PGDM1400	0.228	71.875	56.25	2.639	71.875	3.749	30.208	21.286
1-18+PGT121	0.159	80.208	45.83	2.325	69.792	3.223	34.375	21.429
1-18+10-1074	0.232	83.333	43.75	3.002	69.792	3.187	29.167	24.143
3BNC117+VRC26.25	0.115	80.208	37.50	2.320	67.708	3.154	22.917	24.286
VRC07-523-LS+PGT121	0.164	83.333	44.79	2.928	63.542	3.124	25.000	24.286
PGDM1400+PGT121	0.110	75.000	31.25	2.208	64.583	3.345	17.708	25.286
VRC01+VRC26.25	0.233	69.792	40.62	3.088	63.542	3.311	23.958	27.857
PGDM1400+10-1074	0.165	72.917	29.16	3.049	63.542	3.053	19.792	28.714
VRC26.25+PGT121	0.164	69.792	26.04	2.256	61.458	2.889	20.833	28.857
VRC07-523-LS+10-1074	0.251	82.292	42.70	4.197	60.417	2.762	18.750	29.857
3BNC117+PGT121	0.296	68.750	36.45	3.859	59.375	2.987	20.833	31.286
VRC26.25+10-1074	0.232	70.833	22.91	3.376	54.167	2.555	20.833	32.429
3BNC117+10-1074	0.456	71.875	35.41	5.151	62.500	2.827	15.625	32.571
VRC01+PGT121	0.506	57.292	39.58	5.337	55.208	2.904	17.708	33.000
VRC01+10-1074	0.819	56.250	36.45	7.097	55.208	2.723	17.708	34.286

Table S3. Triple bnAb neutralization characteristics and ranking, Related to Figure 3, and STAR Methods

bnAb	IC80 geometric mean (µg/ml)	IC80 < 1µg/ml breadth	IC80 breadth with 2 bnAbs active	IC99 geometric mean (µg/ml)	IC99 < 10µg/ml breadth (%)	IIP at 30µg/ml (Log10)	IIP > 5Log10 breadth (%)	Average rank
N49P9.6	0.030	94.792	82.29	0.300	93.750	6.516	71.875	2.714
N49P9.6 FR+PGDM1400+10-1074	0.037	95.833	82.29	0.367	93.750	6.303	69.792	3.286
N49P9.6 FR+VRC26.25+PGT121	0.025	95.833	69.79	0.285	93.750	6.173	63.542	5.000
VRC01.23LS+PGDM1400+PGT12	0.052	91.667	84.37	0.514	91.667	5.984	65.625	5.000
N49P9.6 FR+VRC26.25+10-1074	0.031	96.875	70.83	0.345	94.792	6.126	62.500	5.571
N49P9.6+PGDM1400+PGT121	0.054	91.667	84.37	0.585	91.667	5.712	56.250	7.143
VRC01.23LS+PGDM1400+10-	0.068	92.708	84.37	0.638	91.667	5.692	63.542	7.143
N49P9.6+PGDM1400+10-1074	0.071	92.708	84.37	0.723	90.625	5.499	57.292	10.143
N49P9.6+VRC26.25+PGT121	0.049	92.708	71.87	0.622	89.583	5.174	57.292	10.857
VRC01.23LS+VRC26.25+PGT121	0.048	89.583	71.87	0.553	85.417	5.572	61.458	10.857
N6+PGDM1400+PGT121	0.071	88.542	83.33	0.737	86.458	5.921	60.417	12.000
VRC01.23LS+VRC26.25+10-1074	0.063	91.667	72.91	0.685	86.458	5.457	55.208	12.429
1-18+PGDM1400+PGT121	0.056	90.625	78.12	0.677	85.417	5.436	55.208	12.571
N49P9.6+VRC26.25+10-1074	0.065	93.750	72.91	0.763	91.667	5.185	53.125	12.857
VRC07-523-	0.056	89.583	80.20	0.776	87.500	5.337	54.167	13.429
N6+VRC26.25+PGT121	0.066	89.583	71.87	0.759	86.458	5.478	56.250	14.286
N6+PGDM1400+10-1074	0.096	86.458	83.33	0.923	85.417	5.636	59.375	16.571
1-18+PGDM1400+10-1074	0.073	90.625	78.12	0.832	84.375	5.117	51.042	17.571
1-18+VRC26.25+PGT121	0.054	88.542	68.75	0.735	83.333	5.164	53.125	17.857
N6+VRC26.25+10-1074	0.089	87.500	72.91	0.945	87.500	5.251	54.167	18.857
3BNC117+PGDM1400+PGT121	0.070	88.542	73.95	0.938	84.375	4.800	46.875	19.286
VRC07-523-LS+PGDM1400+10-	0.075	88.542	80.20	0.993	84.375	5.068	51.042	19.286
1-18+VRC26.25+10-1074	0.071	89.583	69.79	0.910	83.333	4.818	46.875	20.429
VRC07-523-	0.053	88.542	68.75	0.878	79.167	4.775	46.875	20.429
3BNC117+PGDM1400+10-1074	0.093	87.500	72.91	1.187	83.333	4.738	45.833	23.857
3BNC117+VRC26.25+PGT121	0.064	88.542	63.54	1.004	79.167	4.589	41.667	24.143
VRC07-523-LS+VRC26.25+10-	0.070	88.542	69.79	1.144	77.083	4.559	42.708	24.143
3BNC117+VRC26.25+10-1074	0.085	88.542	63.54	1.283	80.208	4.248	34.375	26.857
VRC01+PGDM1400+PGT121	0.103	81.250	66.66	1.378	80.208	4.767	45.833	27.000
VRC01+PGDM1400+10-1074	0.144	81.250	66.66	1.764	77.083	4.557	42.708	29.000
VRC01+VRC26.25+PGT121	0.107	78.125	58.33	1.424	75.000	4.493	40.625	30.714
VRC01+VRC26.25+10-1074	0.149	78.125	59.37	1.825	73.958	4.144	41.667	31.286

Table S4. N49P9.6-FR polyreactivity/autoreactivity testing, Related to STAR methods

Test	N49P9.6-FR concentration	Reference range	Test result
ANA (Hep2)	25ug/ml	<1:80 = negative	<1:80
Centromere Ab IgG	25ug/ml	29 AU/ml or less=	6 AU/ml
Jo-1 Ab	25ug/ml	29 AU/ml or less= negative	0 AU/ml
SSA-52 (Ro52) IgG	25ug/ml	29 AU/ml or less= negative	0 AU/ml
SSA-60 (Ro60) IgG	25ug/ml	29 AU/ml or less= negative	0 AU/ml
SSB (La) IgG	25ug/ml	29 AU/ml or less= negative	0 AU/ml
Smith (ENA) IgG	25ug/ml	29 AU/ml or less= negative	0 AU/ml
Smith/RNP (ENA) IgG	25ug/ml	29 AU/ml or less= negative	0 AU/ml

Table S5. Individual data for in vivo experiments, Related to Figure 4 and STAR Methods.

Dose	Week 1 viral load	Week 2 viral load (copies/ml)	Week 3 viral load (copies/ml)
**hCD34-NSG-SGM3 Experiment**			
20mg/kg N49P9.6-FR-LS	<	<	<
20mg/kg N49P9.6-FR-LS	2.77E+04	1.09E+06	1.09E+06
20mg/kg N49P9.6-FR-LS	<	<	<
20mg/kg N49P9.6-FR-LS	<	<	<
20mg/kg N49P9.6-FR-LS	<	<	<
20mg/kg N49P9.6-FR-LS	<	<	<
20mg/kg N49P9.6-FR-LS	<	5.61E+05	1.01E+05
10mg/kg N49P9.6-FR-LS	<	<	<
10mg/kg N49P9.6-FR-LS	5.50E+03	Not done (found dead)	Not done (found dead)
10mg/kg N49P9.6-FR-LS	<	<	<
10mg/kg N49P9.6-FR-LS	<	<	<
10mg/kg N49P9.6-FR-LS	<	5.69E+04	6.22E+04
10mg/kg N49P9.6-FR-LS	<	8.42E+03	5.42E+04
10mg/kg N49P9.6-FR-LS	<	<	<
5mg/kg N49P9.6-FR-LS	<	5.24E+04	2.95E+04
5mg/kg N49P9.6-FR-LS	<	<	<
5mg/kg N49P9.6-FR-LS	<	<	<
5mg/kg N49P9.6-FR-LS	<	3.42E+03	2.27E+04
5mg/kg N49P9.6-FR-LS	<	2.51E+05	4.88E+04
5mg/kg N49P9.6-FR-LS	1.93E+02	4.47E+04	1.92E+04
5mg/kg N49P9.6-FR-LS	1.34E+03	7.26E+04	5.13E+04
2mg/kg N49P9.6-FR-LS	<	<	<
2mg/kg N49P9.6-FR-LS	<	5.56E+02	1.47E+04
2mg/kg N49P9.6-FR-LS	6.76E+02	5.13E+04	7.11E+04
2mg/kg N49P9.6-FR-LS	<	<	<
2mg/kg N49P9.6-FR-LS	4.71E+02	3.25E+04	1.83E+04
2mg/kg N49P9.6-FR-LS	<	7.03E+04	4.74E+04
2mg/kg N49P9.6-FR-LS	1.59E+04	9.14E+04	5.44E+04
10mg/kg Synagis	<	5.40E+05	5.40E+05
10mg/kg Synagis	4.67E+02	6.12E+04	9.06E+04
10mg/kg Synagis	6.29E+03	7.04E+04	6.78E+04
10mg/kg Synagis	5.09E+04	9.73E+04	7.73E+04
10mg/kg Synagis	1.25E+05	8.77E+04	3.54E+04
10mg/kg Synagis	3.00E+04	1.62E+05	5.32E+04
10mg/kg Synagis	1.52E+05	6.65E+04	5.68E+04
**hCD34-NSG-IL15 Experiment**			
20mg/kg N49P9.6-FR-LS	<	<	<
20mg/kg N49P9.6-FR-LS	<	<	<
20mg/kg N49P9.6-FR-LS	<	<	<
20mg/kg N49P9.6-FR-LS	<	<	<
20mg/kg N49P9.6-FR-LS	<	<	<
20mg/kg N49P9.6-FR-LS	<	<	<
20mg/kg N49P9.6-FR-LS	<	<	<
10mg/kg N49P9.6-FR-LS	<	<	<
10mg/kg N49P9.6-FR-LS	<	<	<
10mg/kg N49P9.6-FR-LS	<	<	<
10mg/kg N49P9.6-FR-LS	<	<	<
10mg/kg N49P9.6-FR-LS	<	<	<
10mg/kg N49P9.6-FR-LS	4.11E+03	6.92E+04	4.10E+04
10mg/kg N49P9.6-FR-LS	<	<	<
2.5mg/kg N49P9.6-FR-LS	<	<	<
2.5mg/kg N49P9.6-FR-LS	<	<	<
2.5mg/kg N49P9.6-FR-LS	<	<	<
2.5mg/kg N49P9.6-FR-LS	<	<	<
2.5mg/kg N49P9.6-FR-LS	<	<	<
2.5mg/kg N49P9.6-FR-LS	<	<	<
2.5mg/kg N49P9.6-FR-LS	<	<	<
2.5mg/kg N49P9.6-FR-LS	<	2.34E+04	2.50E+04
1.5mg/kg N49P9.6-FR-LS	<	<	<
1.5mg/kg N49P9.6-FR-LS	<	<	<
1.5mg/kg N49P9.6-FR-LS	<	<	<
1.5mg/kg N49P9.6-FR-LS	<	3.46E+04	9.66E+04
1.5mg/kg N49P9.6-FR-LS	<	<	<
1.5mg/kg N49P9.6-FR-LS	1.03E+03	2.46E+04	1.94E+04
1.5mg/kg N49P9.6-FR-LS	<	<	<
10mg/kg Synagis	<	1.88E+04	2.63E+04
10mg/kg Synagis	4.41E+03	6.83E+04	3.12E+04
10mg/kg Synagis	<	3.75E+04	3.20E+04
10mg/kg Synagis	<	3.12E+04	2.31E+04
10mg/kg Synagis	<	<	2.96E+04
10mg/kg Synagis	9.29E+03	6.80E+04	4.45E+04
10mg/kg Synagis	<	2.23E+04	3.95E+04

Table S6. CryoEM Data collection and refinement statistics, Related to Figure S6 and STAR methods

BG505 SOSIP.664-PGT121-N49P9.6-FR complex		
	Holey grid	Holey grid with 2 nm carbon film
**Data Collection**		
Microscope	FEI Glacios	FEI Glacios
Voltage (kV)	200	200
Total exposure dose ($e^-/Å^2$)	54.9633	58.8084
Detector	Gatan K3	Gatan K3
Pixel Size (Å)	0.8893	0.8893
Defocus Range (µm)	0.5-2.7	0.5-2.7
Magnification	45,000	45,000
**Reconstruction**		
Software	CryoSPARC	CryoSPARC
Micrographs collected	1,900	2,468
Number particles extracted/final	1,291,812 / 48,631	1,859,611 / 83,424
Symmetry	C3	C3
Box size (pix)	256	256
Resolution (Å) (FSC 0.143)	4.02	
**Refinement (Phenix) & validation**		
Protein residues	3,144	
Chimera CC	0.71	
EMRinger Score		
Bond lengths (Å)	0.006	
Bond angles (˚)	1.04	
Molprobity score	2.17	
Clash score	13.5	
Rotamer outliers (%)	0.11	
Ramachandran		
Favored (%)	90.7	
Disallowed (%)	0.3	
EMDB	EMD-26648	
PDB	7UOJ	

Table S7. Changes to the BG505 SOSIP.664 trimer upon binding to CD4bs mAbs that contact the adjacent gp120 protomer, Related to Figure 5 and STAR Methods.

Trimer/mAb	a (Å)	b (Å)	c (Å)	d (Å)	e (Å)	f (Å)	Rotation	Rotation (average)
BG505 SOSIP.664/ N49P9.6-FR	46.76	46.65	46.72	56.67	56.80	56.59	1.65, 1.48, 1.56	1.5
BG505 SOSIP.664 (4ZMJ)	45.31	45.33	45.38	54.48	54.48	54.48	0, 0, 0	0
BG505 SOSIP.664/ VRC03 (6CDI)	46.70	46.65	46.63	55.63	55.64	55.50	2.81, 2.87, 2.89	2.9
BG505 SOSIP.664/ N49P6 (6OZ4))	46.03	46.04	46.10	54.45	54.46	54.46	1.07, 1.07, 1.07	1.1
BG505 SOSIP.664/ VRC01-FR (6NNF)	45.90	45.91	45.97	55.25	5.25	55.25	0.724, 0.722, 0.721	0.72
BG505 SOSIP.664/ N6-FR (6NM6)	45.61	45.82	45.70	55.35	55.35	55.35	1.53, 0.848, 0.667	0.76
BG505 SOSIP.664/ CD4 (5THR)	49.76	50.84	51.16	65.60	65.49	65.55	63.8, 65.1, 63.7	64.4

The degree of 'trimer opening' is calculated as described in [2] and defined as the change in position of gp120 relative to gp41 of BG505 SOSIP.664 bound to antibody as compared to unliganded, apo BG505 SOSIP.664 (PDB ID: 4ZMJ[3]). The relative position for each gp120 in the trimer is calculated based on the α-carbon position for residue 375 at the base of the CD4 Phe43 binding pocket relative to the gp41 trimer center (calculated for all trimers aligned based on the α-carbon positions of the central gp41 α7 helices). The distances between Centr and the $^{375}C_{\alpha}$ of each protomer (a, b, c) and the $^{375}C_{\alpha}$ atoms of neighboring protomers (d, e, f) are shown to indicate the extent of the protomer rearrangement relative to gp41. The clockwise rotations of the gp120 subunits are calculated as angles relative to apo BG505 SOSIP.664. The BG505 SOSIP.664 bound to CD4 (PDB: 5THR[4]) is shown as a reference to an 'open' CD4-triggered conformation of trimer.

Table S8. Viral neutralization and expression yield for N49P9.6-FR antibody designs, Related to Figure S9 and STAR Methods.

Design[1]	Approach[2]	Median IC50 (µg/ml)[3]	IC50 fold change[4]	Yield (µg/ml)[5]
N49P9.6-FR (wild type)	-	0.022	-	13.2
H_S60A	Glycan site mutation	**0.013**	**1.7**	13.0
H_H1Q	Rational design	0.033	0.7	13.0
H_A16D	Rational design	0.022	1.0	**18.5**
H_W61R	Rational design	0.023	1.0	**16.1**
L_I32S	Rational design	0.037	0.6	**17.3**
L_T70D	Rational design	**0.012**	**1.8**	8.0
L_FDDK49YSGST	CDRL2 swap	7.1	0.003	6.2
L_I32S_FDDK49YSGST	Rational design, CDRL2 swap	17	0.001	8.6
H_P76aY	FR3 loop design	**0.017**	**1.3**	7.0
H_W76fR	FR3 loop design	0.054	0.4	**52.2**
H_V110T,L_T108Q	V-C hinge consensus	**0.009**	**2.4**	**18.3**

Improved measured properties for designs versus wild-type are shown in bold.

[1]Designs are annotated by chain (H: heavy, L: light) and residue substitution. Substitution FDDK49YSGST corresponds to a CDRL2 residue swap with the VRC07 antibody.

[2]Design approach utilized. Rational design corresponds to germline or consensus residue changes assessed using the Therapeutic Antibody Profiler [5] and Rosetta [6] tools. FR3 design corresponds to structure-based mutagenesis of FR3 loop residues using Rosetta. The V-C consensus design corresponds to simultaneous substitution of variable-constant domain junction residues to consensus residues from related antibodies.

[3]Median IC50 calculated based on neutralization with a global panel of 21 HIV viruses.

[4]Fold change of IC50 compared to wildtype.

[5]Expression yield for 50 ml transfectant.

Table S9. Final variant set showing biophsyical characterization analysis, Related to Figures S11-12 and STAR Methods.

		Conformational Stability						Colloidal Stability							
Molecule	Titer	DSF WSS	DSF T1	DSF T2	Thermal Hold	SEC Δ% Aggregation after low pH	Gdn Inflection Pt 1 (M)	SINS	Viscosity at 170mg/mL	PEG % Protein Recovery	Zenix Main Peak RT	Poly - CL	Poly - Insulin	Poly - dsDNA	Poly - KLH
N49P9.6-FR-LS	0.75	14.67	66.57	none	1.71	0.2	2.07	567	197	38%	10.1	0.03	0.93	0.32	0.57
N49P9.6-FR-LS_GL	0.93	15.43	66.42	none	1.67	0.2	2.14	568	181	35%	9.9	0.04	0.62	0.24	0.26
M-5809	0.59	6.90	70.39	none	1.86	0.2	2.11	564	155	56%	10.2	0.04	0.44	0.00	0.39
M-5809_GL	0.82	7.38	70.12	none	1.81	0.3	2.10	563	100	63%	10.0	0.04	0.57	0.26	0.23
M-5817	0.66	14.81	66.81	none	1.43	-0.1	1.92	567	124	54%	10.2	0.03	0.28	0.00	0.08
M-5817_GL	0.74	16.14	66.31	70.43	1.30	0.2	1.89	559	89	59%	9.9	0.03	0.75	0.33	0.37

Conditional formating was applied relative to the lowest and highest values within the set for columns 1-5 and 8-10. Columns 6, 7, and 11-15 were conditionally formated to acceptable ranges to illustrate that all molecules in the set were not significantly different. Green colors indiate favorable results, red unfavoratble and yellow moderate.

References

1. Wickham, H. (2016). ggplot2: Elegant Graphics for Data Analysis (Springer Publishing Company, Incorporated).
2. Tolbert, W.D., Nguyen, D.N., Tehrani, Z.R., Sajadi, M.M., and Pazgier, M. (2021). Near-Pan-neutralizing, Plasma Deconvoluted Antibody N49P6 Mimics Host Receptor CD4 in Its Quaternary Interactions with the HIV-1 Envelope Trimer. mBio *12*, e0127421. 10.1128/mBio.01274-21.
3. Kwon, Y.D., Pancera, M., Acharya, P., Georgiev, I.S., Crooks, E.T., Gorman, J., Joyce, M.G., Guttman, M., Ma, X., Narpala, S., et al. (2015). Crystal structure, conformational fixation and entry-related interactions of mature ligand-free HIV-1 Env. Nat Struct Mol Biol *22*, 522-531. 10.1038/nsmb.3051.
4. Wang, H., Cohen, A.A., Galimidi, R.P., Gristick, H.B., Jensen, G.J., and Bjorkman, P.J. (2016). Cryo-EM structure of a CD4-bound open HIV-1 envelope trimer reveals structural rearrangements of the gp120 V1V2 loop. Proc Natl Acad Sci U S A *113*, E7151-E7158. 10.1073/pnas.1615939113.
5. Raybould, M.I.J., Marks, C., Krawczyk, K., Taddese, B., Nowak, J., Lewis, A.P., Bujotzek, A., Shi, J., and Deane, C.M. (2019). Five computational developability guidelines for therapeutic antibody profiling. Proc Natl Acad Sci U S A *116*, 4025-4030. 10.1073/pnas.1810576116.
6. Leman, J.K., Weitzner, B.D., Lewis, S.M., Adolf-Bryfogle, J., Alam, N., Alford, R.F., Aprahamian, M., Baker, D., Barlow, K.A., Barth, P., et al. (2020). Macromolecular modeling and design in Rosetta: recent methods and frameworks. Nat Methods *17*, 665-680. 10.1038/s41592-020-0848-2.
